# Supplementary material for: Prognostic implications of left ventricular strain by speckle-tracking echocardiography in population-based studies: a systematic review protocol of the published literature
Source: BMJ Open. 2018 Jul 16;8(7):e023346. doi: 10.1136/bmjopen-2018-023346 (PMC6082461; doi:10.1136/bmjopen-2018-023346)
Supplement: Supplementary file 1 [file bmjopen-2018-023346supp001.pdf]

Supplementary file 1. Databases' search strategy

**MEDLINE**	Database: Ovid MEDLINE(R) Epub Ahead of Print, In-Process & Other Non-Indexed Citations, Ovid MEDLINE(R) Daily and Ovid MEDLINE(R) <1946 to Present><br>Search Strategy:<br>--<br>1 Ventricular Function, Left/ (32622)<br>2 Ventricular Dysfunction, Left/ (24705)<br>3 left ventric*.mp. (179585)<br>4 LV.mp. (41504)<br>5 1 or 2 or 3 or 4 (198982)<br>6 Echocardiography/ (78179)<br>7 speckle tracking.mp. (3722)<br>8 STE.mp. (1905)<br>9 strain.mp. (385200)<br>10 deformation.mp. (36853)<br>11 mechanic*.mp. (390628)<br>12 torsion.mp. (22257)<br>13 twist.mp. (10298)<br>14 rotation.mp. (95760)<br>15 GLS.mp. (1508)<br>16 6 or 7 or 8 or 9 or 10 or 11 or 12 or 13 or 14 or 15 (963614)<br>17 Cardiovascular Diseases/ (128863)<br>18 cardiovascular disease*.mp. (220948)<br>19 Heart Failure/ (103286)<br>20 Heart failure.mp. (176882)<br>21 HF.mp. (35998)<br>22 Mortality/ (39168)<br>23 mortality.mp. (670200)<br>24 Death/ (16230)<br>25 death.mp. (691814)<br>26 Morbidity/ (27417)<br>27 morbidity.mp. (330859)<br>28 (cardi* adj3 (event* or outcome*)).mp. (66991)<br>29 17 or 18 or 19 or 20 or 21 or 22 or 23 or 24 or 25 or 26 or 27 or 28 (1690357)<br>30 predict*.mp. (1382430)<br>31 prognos*.mp. (734497)<br>32 30 or 31 (1934125)<br>33 exp Cohort Studies/ (1714168)<br>34 (cohort adj (study or studies)).mp. (310492)<br>35 (Follow up adj (study or studies)).mp. (604740)<br>36 Longitudinal.mp. (250750)<br>37 (observational adj (study or studies)).mp. (109821)<br>38 Epidemiologic studies/ (7611)

	39 (epidemiologic* adj (study or studies)).mp. (79971)<br>40 population based study.mp. (24755)<br>41 general population.mp. (85498)<br>42 communit*.mp. (535197)<br>43 33 or 34 or 35 or 36 or 37 or 38 or 39 or 40 or 41 or 42 (2539317)<br>44 5 and 16 and 29 and 32 and 43 (2940)
**EMBASE**	Database: Embase Classic+Embase <1947 to 2018 February 27><br>Search Strategy:<br>--<br>1 heart left ventricle function/ (39176)<br>2 left ventric*.mp. (332499)<br>3 LV.mp. (83461)<br>4 1 or 2 or 3 (353062)<br>5 echocardiography/ (178295)<br>6 exp speckle tracking echocardiography/ (3150)<br>7 speckle tracking.mp. (10536)<br>8 STE.mp. (4416)<br>9 strain.mp. (762166)<br>10 deformation.mp. (42935)<br>11 mechanic*.mp. (498347)<br>12 torsion.mp. (27446)<br>13 twist.mp. (12502)<br>14 rotation.mp. (109923)<br>15 GLS.mp. (3938)<br>16 5 or 6 or 7 or 8 or 9 or 10 or 11 or 12 or 13 or 14 or 15 (1553350)<br>17 cardiovascular disease/ (241943)<br>18 cardiovascular disease*.mp. (336057)<br>19 heart failure/ (212076)<br>20 Heart Failure.mp. (347509)<br>21 HF.mp. (60866)<br>22 mortality/ (722163)<br>23 cardiovascular mortality/ (29486)<br>24 all cause mortality/ (4807)<br>25 mortality.mp. (1271143)<br>26 death/ (284488)<br>27 death.mp. (1097060)<br>28 morbidity/ (309365)<br>29 morbidity.mp. (569914)<br>30 (cardi* adj3 (event* or outcome*)).mp. (110928)<br>31 17 or 18 or 19 or 20 or 21 or 22 or 23 or 24 or 25 or 26 or 27 or 28 or 29 or 30 (2824491)<br>32 predict*.mp. (1798936)<br>33 prognos*.mp. (982782)

	34	32 or 33 (2544622)
	35	longitudinal study/ (109328)
	36	longitudinal.mp. (297876)
	37	prospective study/ (426930)
	38	cohort analysis/ (346132)
	39	(Cohort adj (study or studies)).mp. (217636)
	40	(follow up adj (study or studies)).mp. (63815)
	41	(observational adj (study or studies)).mp. (172984)
	42	(epidemiologic* adj (study or studies)).mp. (100634)
	43	population based study.mp. (33254)
	44	general population.mp. (124629)
	45	communit*.mp. (641283)
	46	35 or 36 or 37 or 38 or 39 or 40 or 41 or 42 or 43 or 44 or 45 (1963334)
	47	4 and 16 and 31 and 34 and 46 (4098)

‘adj3’ is a proximity operator and indicates within three words;

‘OR and AND’ are Boolean operators;

‘*’ indicates truncation;

‘mp.’ means a keyword search of title, abstract, original title, name of substance word, subject heading word and keyword heading word.
