# Supplementary material for: Prognostic implications of left ventricular strain by speckle-tracking echocardiography in population-based studies: a systematic review protocol of the published literature
Source: BMJ Open. 2018 Jul 16;8(7):e023346. doi: 10.1136/bmjopen-2018-023346 (PMC6082461; doi:10.1136/bmjopen-2018-023346)
Supplement: Supplementary file 2 [file bmjopen-2018-023346supp002.pdf]

Eligibility Form: Prognostic Implications of Left Ventricular Strain by Speckle-Tracking Echocardiography in Population-based Studies

**1. Reference details**		
Ref ID		
Reference citation		
First author		
Year of publication		
Title of the paper		
Type of publication	Paper ☐	Abstract ☐
Assessor's identifier	1 ☐	2 ☐
Date		
Contact details of authors		

**2. Study eligibility**				
Inclusion of the study	Yes ☐	No ☐	Unclear ☐	
**Eligibility criteria**	**Reason for exclusion (if excluded)**		Yes	No
Longitudinal (cohort) study	• Ineligible study design e.g. Cross-sectional, case-control, or CRT		☐	☐
General population, community/population-based samples/ unselected community-dwelling individuals	• Ineligible population e.g. patients with established CVD, not population-based, unrepresentative samples of the general population		☐	☐
LV strain measured by STE	• Ineligible exposure e.g. measured by MRI		☐	☐
	• Ineligible outcome (Different from outcomes considered in this review e.g. LV remodelling)		☐	☐
	• Duplicate		☐	☐
	• Reviews, abstracts, letters, or conference proceeding		☐	☐
	• Other (please specify): e.g. not in English or in a peer reviewed journal		☐	☐
Notes:				

**Additional information**
Notes:

DO NOT PROCEED IF PAPER EXCLUDED FROM REVIEW

Data Extraction Form: Prognostic Implications of Left Ventricular Strain by Speckle-Tracking Echocardiography in Population-based Studies

**3. Study details**		
Ref ID		
Study (cohort) name		
Study design	Longitudinal (cohort) study ☐	
Region/country		
Sample size		
Mean (range) age	Mean ± SD :	Range (IQR):
Sex	Male, n (%):	Female, n (%):
Ethnicity		
Follow-up duration		
Clinical variables (n, %)		
• Hypertension		
• Diabetes mellitus		
• Dyslipidaemia		
• Smoking status		
• Known CVD		
Dose the study include/mention a statement regarding the ethical approval, or adherence to an appropriate standard (such as the declaration of Helsinki)? Yes ☐ No ☐		

**4. Exposure details**				
Hardware			Software	
Procedure:	2D-STE ☐	3D-STE ☐		
Measured LV strain (direction)			Images obtained from	number of LV segments
Longitudinal:	Yes ☐	No ☐		
Circumferential:	Yes ☐	No ☐		
Radial:	Yes ☐	No ☐		
Others (specify):	Yes ☐	No ☐		
Number of sonographers used to perform the analysis:			Provide data on intra- or inter-observer variability or both?	Yes ☐ No ☐ Note:
Notes:				

**5. Outcomes' details**	
Type of outcomes used in this review	Outcomes reported in this study (n)
**Primary outcome(s)**<br>All-cause mortality	

**Secondary outcome (s)**<br>A. Composite **cardiovascular** (CV) end point includes: CV mortality, CHD events (MI, unstable angina, angina/ischemia requiring emergent hospitalization or revascularization), HF hospitalization, new onset AF, life threatening arrhythmia , recorded automatic implantable cardioverter defibrillator (AICD) shocks, stroke, transit ischemic attack, peripheral arterial disease with arterial revascularization procedure.	
B. Composite **cardiac** end point includes: CV mortality, CHD events, HF hospitalization, new onset AF, life threatening arrhythmia, recorded AICD shocks.	
Report an individual end point included in the composite cardiac or CV end point	Yes ☐ No ☐<br>List if yes:
Assessment of outcome	☐ independent blind assessment ☐ record linkage<br>☐ self-report ☐ others
Notes:	

**6. Available number of participants**		
		Number
Baseline sample size	Yes ☐ No ☐	
Excluded from the baseline	Yes ☐ No ☐<br>Reason if yes:	
Lost to follow-up	Yes ☐ No ☐	
Other losses	Yes ☐ No ☐	
Total number included in the analysis	Yes ☐ No ☐	
All subjects accounted for	Yes ☐ No ☐	
Notes:		

**7. Statistical analysis**	
Statistical method used	Cox proportional hazards regression Yes ☐ No ☐<br>Others if 'No':
Analysis includes males and	Yes ☐ No ☐

females	
Study reports results by sex	Yes ☐ No ☐
List all models used including unadjusted	

7. **Results of the association of interest**								
Exposure	Outcome	Group (M/F/All)	Total (n) included in the analysis	Events/non-events	Effect estimate	95% CI, SE, p	Unit	Adjustments (none if unadjusted)
