# Supplementary material for: Prognostic implications of left ventricular strain by speckle-tracking echocardiography in population-based studies: a systematic review protocol of the published literature
Source: BMJ Open. 2018 Jul 16;8(7):e023346. doi: 10.1136/bmjopen-2018-023346 (PMC6082461; doi:10.1136/bmjopen-2018-023346)
Supplement: Supplementary file 3 [file bmjopen-2018-023346supp003.pdf]

NEWCASTLE - OTTAWA QUALITY ASSESSMENT SCALE - COHORT STUDIES (Amended)

Note: A study can be awarded a maximum of one star for each numbered item within the Selection and Outcome categories.

Selection

1) Representativeness of the cohort
   a) Truly representative of the average person in the community ❋
   b) Somewhat representative of the average person in the community (sampling described and designed to achieve adequately representative sample of the community)❋
   c) Selected group e.g. nurses, volunteers
   d) No description of the derivation of the cohort
2) Assessment of exposure reliability
   a) Include assessment of inter- and intra-observer variabilities ❋
   b) Include assessment of intra-observer variability ❋
   c) No description
3) Completeness of collection of potential confounders[1](i.e. CVD risk factors)
   a) Complete ❋
   b) Partially complete (≥ 5) ❋
   c) Partially complete (< 5)
   d) No description
4) Demonstration that the outcome of interest not present at start of study (and/or sensitively analysis)
   a) Yes ❋
   b) No

Outcome

1) Assessment of outcome
   a) Independent blind assessment ❋
   b) Record linkage ❋
   c) Self-report
   d) No description
2) Was follow-up long enough for outcomes to occur
   a) yes (an adequate follow up period for outcome of interest is a year) ❋
   b) no
3) Adequacy of follow up of cohorts
   a) Complete follow up - all subjects accounted for ❋
   b) Subjects lost to follow up unlikely to introduce bias - small number lost - > 80% follow up, or description provided of those lost ❋
   c) Follow up rate < 80% and no description of those lost
   d) No statement

The total quality score will be calculated by averaging the score of the two researchers with the range from 0 (lowest quality) to 7 (highest quality) and will be provided as a descriptive variable of the included studies.

---

1 Based on Framingham risk factors modified to include ethnicity: age, sex, blood pressure, antihypertensive medication (yes/no), total cholesterol, HDL cholesterol (or BMI), smoking, diabetes mellitus, and ethnicity.
