# Supplementary material for: Prognostic implications of left ventricular strain by speckle-tracking echocardiography in population-based studies: a systematic review protocol of the published literature
Source: BMJ Open. 2018 Jul 16;8(7):e023346. doi: 10.1136/bmjopen-2018-023346 (PMC6082461; doi:10.1136/bmjopen-2018-023346)
Supplement: Author's manuscript [file bmjopen-2018-023346.draft_revisions.pdf]

BMJ Open is committed to open peer review. As part of this commitment we make the peer review history of every article we publish publicly available.

When an article is published we post the peer reviewers' comments and the authors' responses online. We also post the versions of the paper that were used during peer review. These are the versions that the peer review comments apply to.

The versions of the paper that follow are the versions that were submitted during the peer review process. They are not the versions of record or the final published versions. They should not be cited or distributed as the published version of this manuscript.

BMJ Open is an open access journal and the full, final, typeset and author-corrected version of record of the manuscript is available on our site with no access controls, subscription charges or pay-per-view fees (http://bmjopen.bmj.com).

If you have any questions on BMJ Open's open peer review process please email info.bmjopen@bmj.com

BMJ Open

Prognostic Implications of Left Ventricular Strain by Speckle-Tracking Echocardiography in Population-based Studies: A Systematic Review Protocol of the Published Literature

Journal:	*BMJ Open*
Manuscript ID	bmjopen-2018-023346
Article Type:	Protocol
Date Submitted by the Author:	03-Apr-2018

Complete List of Authors:	Al Saikhan, Lamia; University College London , Institute of Cardiovascular Science ; Imam Abdulrahman Bin Faisal University, Department of Cardiac Technology, College of Applied Medial Sciences<br>Park, Chloe; University College London , Institute of Cardiovascular Science<br>Hardy, Rebecca; University College London, MRC Unit for Lifelong Health and Ageing<br>Hughes, A; University College London, Institute of Cardiovascular Science

Keywords:	Speckle tracking echocardiography, Community dwelling individuals, Left ventricular strain, Mortality, Cardiovascular disease

SCHOLARONE™
Manuscripts

For peer review only

Prognostic Implications of Left Ventricular Strain by Speckle-Tracking Echocardiography in Population-based Studies: A Systematic Review Protocol of the Published Literature

Lamia Al Saikhan[1, 2], Chloe Park[1], Rebecca Hardy[3], Alun Hughes[1]

[1]Institute of Cardiovascular Science, University College London, London, UK

[2]Department of Cardiac Technology, College of Applied Medial Sciences, Imam Abdulrahman Bin Faisal University, Dammam, Kingdom of Saudi Arabia

[3]MRC Unit for Lifelong Health and Ageing, University College London, London, UK.

lamia.saikhan.16@ucl.ac.uk

chloe.park@ucl.ac.uk

rebecca.hardy@ucl.ac.uk

alun.hughes@ucl.ac.uk

Correspondence:

Lamia Khaled Al Saikhan

Institute of Cardiovascular Science

University College London

Gower Street

London WC1E 6BT

lamia.saikhan.16@ucl.ac.uk

Tel: 020 7677 1857

Word count: 2157 (excluding title page, abstract, references, figures and tables)

Keywords: Community dwelling individuals, Speckle tracking echocardiography, Mortality, Cardiovascular disease, Left ventricular strain.

For peer review only

ABSTRACT

Introduction: Left ventricular (LV) strain by speckle-tracking echocardiography (STE) is a comparatively new prognostic marker. Meta-analyses relating LV strain by STE to outcomes have been conducted in selected patient-based populations with established or suspected cardiovascular (CV) diseases. However, the evidence related to population-based studies of unselected community dwelling individuals is uncertain. The aim of this study is to provide a comprehensive systematic review and analysis of the current available literature regarding LV strain by STE as a predictor of adverse outcomes in population-based studies.

Methods and analyses: Thesaurus and text-word searching will be used to search two online databases (MEDLINE and EMBASE) and additional sources will be identified from citation metrics and reference lists' search. Dual search results' screening, data extraction and quality assessment will be performed. Cohort studies of community/population-based samples who have had STE and followed-up longitudinally for mortal and morbid events, and published in English and peer reviewed journals will be included. Primary outcome will be all-cause mortality whereas secondary outcomes will be composite cardiac and CV end points. Risk of bias will be assessed using Newcastle-Ottawa quality assessment scale of cohort studies that will be modified as appropriate. Any arising discrepancies will be discussed and resolved through consensus.

Ethics and dissemination: Ethical approval is not required as this is a protocol for a systematic review. The findings of this study will be presented at scientific conferences and published in a peer reviewed journal. Any amendments to the protocol will be documented and updated in the PROSPERO registry.

Systematic review registration number: CRD42018090302.

For peer review only

For peer review only - http://bmjopen.bmj.com/site/about/guidelines.xhtml

ARTICLE SUMMARY

Strengths and limitations of this study

- This systematic review will evaluate the evidence related to the incremental prognostic value of left ventricular (LV) strain by speckle tracking echocardiography (STE) in relation to mortality and cardiovascular events in unselected community dwelling individuals or population-based samples known to be at low risk relative to selected diseased populations.
- The results of this systematic review will add to the existing evidence on the utility of STE-based LV strain as a measure of cardiac function and risk, and influence its use in longitudinal population-based studies.
- Because of the anticipated heterogeneity in results/presentation of results and/or study design across a limited number of existing studies, there may be a limited scope for meta-analysis. When meta-analysis is possible, heterogeneity in associations between studies may be difficult to explain.

For peer review only - http://bmjopen.bmj.com/site/about/guidelines.xhtml

INTRODUCTION

Cardiovascular disease (CVDs) is the leading cause of mortality worldwide and accounts for 31% of deaths.[1] Assessment of left ventricular (LV) global systolic function is central in evaluating prognosis, as well as in determining therapeutic strategies in CVDs.[2] LV ejection fraction (LVEF) is a simple and widely used parameter that is most commonly measured by echocardiography.[3] Reduced LVEF is known to be associated with unfavorable outcomes in populations with established CVDs,[4-7] but also in people without known CVDs.[8 9] However, this parameter suffers from a number of inherent limitations including load dependency, low reproducibility, and geometric assumptions.[3] Furthermore, its inverse association with outcomes varies considerably across the spectrum of LVEF, being greatest in moderately to severely impaired LV systolic function.[7]

LV strain imaging, tissue Doppler- and speckle tracking- based, has emerged as a powerful and more sensitive tool allowing an accurate quantification of myocardial mechanics including longitudinal, circumferential and radial shortening/lengthening, and torsion.[10] Strain, a dimensionless index, is defined as a change in length of a myocardial segment: strain (ε) $=\Delta L/L_0$, where ΔL=change in length and L_0= original length.[3 10 11] The major limitation of tissue Doppler-derived strain (natural strain) is the angle dependency of measurements and this has been superseded by speckle-tracking echocardiography [STE] (Lagrangian strain).[10] As the term implies, STE is based on the analysis of myocardial features (speckles) on grayscale B-mode images that are generated by ultrasound interference patterns within the myocardium.[10-12] These speckles are tracked frame by frame during the cardiac cycle providing a sensitive and objective measure of global and regional myocardial function (i.e. Lagrangian strain).[10-12] Unlike tissue Doppler imaging (TDI), STE is angle independent and can differentiate active from passive motion,[13] and has been validated in an

For peer review only - http://bmjopen.bmj.com/site/about/guidelines.xhtml

experimental setting against sonomicrometry[14] as well as in a clinical setting against cardiac magnetic resonance imaging (CMR).[15]

STE-based LV strain imaging has been shown to have an independent and additive prognostic value over conventional echocardiographic measures in a number of studies of diseased populations. These included patients with chronic[16 17] or acute[18] heart failure (HF) including both people with reduced[19 20] and preserved[21] LVEF. Further, STE-based LV strain imaging has prognostic utility after acute myocardial infarction (MI)[22 23] and in chronic ischemic cardiomyopathy.[24] More importantly, STE-based LV strain especially global longitudinal strain (GLS) has a potential value in detecting subclinical left ventricular systolic dysfunction (LVSD) when LVEF is within normal limits.[3] Indeed, the most common clinical setting is the prediction of cardiotoxicity in patients receiving cancer thereby.[25] GLS has also shown an association with unfavorable outcomes in asymptomatic severe aortic stenosis[26] and hypertrophic cardiomyopathy[27] with preserved LVEF. Alterations in GLS despite preserved LVEF have been demonstrated in populations with risk factors that predispose to CVDs including aging[28], diabetes mellitus (DM)[29], hypertension[30] and obesity[31], and may be the earliest marker of LVSD.

Previous systematic reviews and meta-analyses relating LV strain by STE to outcomes have been conducted in selected patient-based samples with established[32-35] or established plus suspected CVDs.[36] However, no systematic review has been performed of studies which have recruited unselected community dwelling individuals or population-based samples, who are known to be at low risk compared to selective diseased populations, and the utility of STE in this setting is uncertain. LV strain could identify people from the general population who may be at risk of cardiovascular (CV) mortality and morbidity independent of conventional risk factors. We, therefore, hypothesized that LV strain by STE provides additive prognostic value in unselected community-dwelling individuals. The main aim of

For peer review only

this study is to carry out a systematic review and, where possible, meta-analysis of the current literature relating LV strain by STE to mortality and CV morbidity in the general population.

For peer review only

For peer review only - http://bmjopen.bmj.com/site/about/guidelines.xhtml

MEFTHODS

Protocol registration

The protocol of this review is registered with the PROSPERO database (systematic review registration number: CRD42018090302).

Reporting

This systematic review will be conducted and reported in adherence with Preferred Reporting Items for Systematic Reviews and Meta-Analysis (PRISMA) statement[37] and PRISMA-protocols.[38]

Eligibility criteria

Inclusion criteria

The inclusion criteria which relate to the type of study design, population, and measurement procedure are summarized in table 1. Studies will only be included if they assess the prospective association of LV strain with at least one of the pre-specified outcomes in unselected community-dwelling individuals (table 1).

For peer review only - http://bmjopen.bmj.com/site/about/guidelines.xhtml

Table 1 Eligibility criteria of the systematic review	
Type of Study design	- longitudinal (cohort) studies
Type of participants/population	- Adults (>18 years)<br>- General population, community/population-based samples , or unselected community-dwelling individuals
Type of procedure	- Speckle tracking echocardiography (STE)
Measured parameters (exposure)	- LV strain measured by STE in any direction of the myocardial motion (i.e. global longitudinal strain and/or circumferential strain and/or radial strain and/or transverse strain, and/or any STE derived parameters, e.g. torsion and/or twist)
Type of outcomes	- Primary outcome: All-cause mortality<br>- Secondary outcomes: Composite cardiovascular and cardiac end points (see text for details)
Year of publication	- No limit applied
Languages	- Articles published in English
Publication status	- Published in a peer-reviewed journal

Outcomes

The primary outcome will be all-cause mortality. Secondary outcomes will be (a) composite CV end points, including any combination of CV mortality, coronary heart disease (CHD) events (MI, unstable angina, angina/ischemia requiring emergent hospitalization or revascularization), HF hospitalization, new onset atrial fibrillation (AF), life threatening arrhythmia, recorded automatic implantable cardioverter defibrillator (AICD) shocks, stroke, transit ischemic attack, or peripheral arterial disease with arterial revascularization procedure, or (b) composite cardiac end point, including any combination of CV mortality, CHD events (MI, unstable angina, angina/ischemia requiring emergent hospitalization, or revascularization), HF hospitalization, new onset AF, life threatening arrhythmia, recorded AICD shocks. Tertiary outcomes will be any individual secondary end point included in the composite cardiac or CV end point.

For peer review only - http://bmjopen.bmj.com/site/about/guidelines.xhtml

For peer review only

Exclusion criteria

The exclusion criteria will be as follows: (1) studies not meeting the inclusion criteria; (2) case-control studies, cross-sectional studies, or randomized controlled trials; (3) studies in unrepresentative samples of the general population (e.g. clinic populations) or patients with established/known CVDs (e.g. patients with MI or HF).; (4) LV strain measured by TDI or by another imaging modality (e.g. CMR); (5) studies with end points that do not match those specified in this review (e.g. LV remodeling); or (6) abstracts, reviews, conference preceding or letters to the editorial.

Search Strategy

Electronic searches

This literature search will be performed systematically in the following online databases: MEDLINE (1946 to present) and EMBASE Classic + EMBASE (1947 to present) via OvidSP interface. A Combination of thesaurus and text-word searching will be used to comprehensively extract all relevant articles. Boolean operators (AND/OR), proximity operators and truncation commands will be used when necessary to narrow the search to the relevant literature. The effectiveness of the search stagey will be tested and refined accordingly. The relevant thesaurus terms will be developed first for the MEDLINE database and will be checked and modified for EMBASE database as appropriate. A modified version of SIGN filters for observational studies will be used to capture the required studies (i.e. population –based studies only). No time or language limit will be applied to the search and the final date on which the search will be carried out will be stated. Databases' search strategy is shown in online supplementary file 1.

Additional sources

For peer review only - http://bmjopen.bmj.com/site/about/guidelines.xhtml

Other sources will be identified by thoroughly searching the reference lists of all relevant articles. We will also search the citation metrics of those articles using Web of Science Core Collection for additional studies.

DATA COLLECTION AND ANALYSES

Study selection

The search results from each database will be retrieved and merged using reference manger software (i.e. in a single EndNote library). Duplicates will be identified using the command 'Find Duplicates' and will be removed before screening is commenced. Titles and abstracts will then be screened for eligibility by two researchers working independently and irrelevant articles will be dismissed. Full-texts of potentially eligible articles will be retrieved and double screened. A predefined eligibility form (see supplementary file 2) will be applied to the identified studies to determine whether the inclusion criteria are met. Reasons for exclusions will be recorded. Discrepancies will be reviewed and resolved through consensus of all reviewers. The study selection process will be summarized using PRISMA flow diagram (figure 1).

Data extraction

We will use a predefined data extraction form (see supplementary file 2) which will be piloted on a sample of included studies to ensure that all relevant information is captured. Data will be extracted by two researchers independent of each other and any discrepancies will be discussed in order to achieve consensus. The following data will be extracted: (1) citation details including title, type and year of publication; (2) study and participant details including name, region and design of the study, sample size, demographics (age, sex ethnicity), and follow up duration; (3) clinical variables including the presence or absence of

For peer review only - http://bmjopen.bmj.com/site/about/guidelines.xhtml

hypertension, DM, dyslipidemia, smoking status and known CVDs; (4) exposure details including the hardware and the software used for the acquisition and the analysis as well as details regarding the measured LV strain including the direction of the analyzed myocardial motion, the echocardiographic images used for the analysis and the number of LV segments involved.; (5) outcomes' details (i.e. all-cause mortality, composite CV and cardiac end points) including how these were ascertained; (6) statistical methods used e.g. cox proportional hazards regression as well as statistics related to the association of interest e.g. hazard (or rate) ratio, related measures of precision (i.e. 95% confidence interval or standard error) and statistical significance (P-value); and (7) information related to adjustment for potential confounders.

Quality assessment

Quality of included studies will be assessed using a pre-assigned scale (Newcastle-Ottawa quality assessment scale of cohort studies)[39] that will be modified according to the review question to capture all relevant sources of potential bias (see supplementary file 3).[40] Two reviewers will judge the quality of each study by considering the following criteria**:** Representativeness of the cohort, completeness of collection of potential confounders, follow-up duration, assessment of outcome and the reliability of exposure (i.e. LV strain). The total quality score will be reported as the average of the two researchers' scores ranging from 0, the lowest quality score, to 7, the highest quality score. Studies will be included irrespective of the quality assessment, but sensitivity analyses will be carried out to test the impact of removing the studies with the lowest quality score one at a time.

This systematic review does not study a treatment comparison, and hence quality of evidence assessment based on strict GRADE guidelines (i.e. Grading Quality of Evidence and Strength of Recommendations) is not directly applicable. However, we will employ

For peer review only

relevant components of a GRADE evaluation, namely quality of study, consistency and effect size in evaluating the strength of evidence; the issue of generalizability is dealt with through the inclusion/exclusion criteria.

Data synthesis

Descriptive summary of the characteristics and findings of the included studies will be provided in tables. We anticipate that there may be a limited scope for meta-analysis due to differences in the method of analysis and the reporting of results across studies. If the included studies are sufficiently homogenous and report the same statistics (e.g. comparable hazard ratios), a quantitative synthesis using random effect meta-analysis will be used to pool the results of the association of interest. Based on our expectation, we will likely to use the hazard ratio and 95% confidence interval as an effect estimate for presenting the results, and will present the data graphically in forest plots.

The degree of heterogeneity will be assessed using Higgins Thompson I^2 test[41 42] and Cochran's Q test.[43] If data allow, we will perform subgroup analyses or meta-regression to explore pre-specified potential sources of between-study heterogeneity: a) study quality, b) variation in exposure measurement (e.g. vendors), and c) variation in CV and cardiac outcomes definition. Although it may not be possible, we plan to do subgroup analyses by sex and age categories (<65 and ≥65 years). Possible publication bias will be assessed using a Funnel plot.[44]

For peer review only - http://bmjopen.bmj.com/site/about/guidelines.xhtml

DISCUSSION

This systematic review will identify and synthesize evidence regarding STE-derived measures as a prognostic indicators of mortality and CV events in unselected community dwelling individuals. This will add to evidence on the utility of STE as a measure of cardiac function and risk, and influence its use (or otherwise) in longitudinal population-based studies.

ETHICS AND DISSEMINATION:

This protocol is for conducting a systematic review and hence no ethical approval is required. The findings of this review will be presented at scientific conferences and published in a peer-reviewed journal.

For peer review only

Author Statement /Contributors

LA is the guarantor of the review who drafted the protocol and registered it in PROSPERO. LA and AH contributed in developing the eligibility criteria, search strategy, and data extraction and quality assessment strategy. AH and CP reviewed and amended the protocol. RH provided statistical advice and reviewed and commented on the protocol.

Funding statement None

Competing interest statement None declared

Provenance and peer review Not commissioned; externally peer reviewed.

For peer review only - http://bmjopen.bmj.com/site/about/guidelines.xhtml

REFERNCES

1. WHO. Cardiovascular diseases (CVDs) [Internet]. WHO [updated May 2017. Available from: http://www.who.int/mediacentre/factsheets/fs317/en/. accessed 12 March 2018.
2. Ponikowski P, Voors AA, Anker SD, et al. 2016 ESC Guidelines for the diagnosis and treatment of acute and chronic heart failure: The Task Force for the diagnosis and treatment of acute and chronic heart failure of the European Society of Cardiology (ESC)Developed with the special contribution of the Heart Failure Association (HFA) of the ESC. *Eur Heart J* 2016;37:2129-200.
3. Potter E, Marwick TH. Assessment of Left Ventricular Function by Echocardiography: The Case for Routinely Adding Global Longitudinal Strain to Ejection Fraction. *JACC Cardiovasc Imaging* 2018;11:260-74.
4. Cohn JN, Johnson GR, Shabetai R, et al. Ejection fraction, peak exercise oxygen consumption, cardiothoracic ratio, ventricular arrhythmias, and plasma norepinephrine as determinants of prognosis in heart failure. The V-HeFT VA Cooperative Studies Group. *Circulation* 1993;87:VI5-16.
5. Gomes JA, Mehta D, Ip J, et al. Predictors of long-term survival in patients with malignant ventricular arrhythmias. *Am J Cardiol* 1997;79:1054-60.
6. Gottdiener JS, McClelland RL, Marshall R, et al. Outcome of congestive heart failure in elderly persons: influence of left ventricular systolic function. The Cardiovascular Health Study. *Ann Intern Med* 2002;137:631-9.
7. Curtis JP, Sokol SI, Wang Y, et al. The association of left ventricular ejection fraction, mortality, and cause of death in stable outpatients with heart failure. *J Am Coll Cardiol* 2003;42:736-42.
8. Hays AG, Sacco RL, Rundek T, et al. Left ventricular systolic dysfunction and the risk of ischemic stroke in a multiethnic population. *Stroke* 2006;37:1715-9.
9. Yeboah J, Rodriguez CJ, Stacey B, et al. Prognosis of individuals with asymptomatic left ventricular systolic dysfunction in the multi-ethnic study of atherosclerosis (MESA). *Circulation* 2012;126:2713-9.
10. Mor-Avi V, Lang RM, Badano LP, et al. Current and evolving echocardiographic techniques for the quantitative evaluation of cardiac mechanics: ASE/EAE consensus statement on methodology and indications endorsed by the Japanese Society of Echocardiography. *J Am Soc Echocardiogr* 2011;24:277-313.
11. Nesbitt GC, Mankad S, Oh JK. Strain imaging in echocardiography: methods and clinical applications. *Int J Cardiovasc Imaging* 2009;25 Suppl 1:9-22.
12. Mondillo S, Galderisi M, Mele D, et al. Speckle-tracking echocardiography: a new technique for assessing myocardial function. *Journal of ultrasound in medicine : official journal of the American Institute of Ultrasound in Medicine* 2011;30:71-83.
13. Collier P, Phelan D, Klein A. A Test in Context: Myocardial Strain Measured by Speckle-Tracking Echocardiography. *J Am Coll Cardiol* 2017;69:1043-56.
14. Langeland S, D'Hooge J, Wouters PF, et al. Experimental validation of a new ultrasound method for the simultaneous assessment of radial and longitudinal myocardial deformation independent of insonation angle. *Circulation* 2005;112:2157-62.
15. Amundsen BH, Helle-Valle T, Edvardsen T, et al. Noninvasive myocardial strain measurement by speckle tracking echocardiography: validation against

For peer review only - http://bmjopen.bmj.com/site/about/guidelines.xhtml

sonomicrometry and tagged magnetic resonance imaging. *J Am Coll Cardiol* 2006;47:789-93.

16. Nahum J, Bensaid A, Dussault C, et al. Impact of longitudinal myocardial deformation on the prognosis of chronic heart failure patients. *Circ Cardiovasc Imaging* 2010;3:249-56.
17. Zhang KW, French B, May Khan A, et al. Strain improves risk prediction beyond ejection fraction in chronic systolic heart failure. *J Am Heart Assoc* 2014;3:e000550.
18. Cho GY, Marwick TH, Kim HS, et al. Global 2-dimensional strain as a new prognosticator in patients with heart failure. *J Am Coll Cardiol* 2009;54:618-24.
19. Mignot A, Donal E, Zaroui A, et al. Global longitudinal strain as a major predictor of cardiac events in patients with depressed left ventricular function: a multicenter study. *J Am Soc Echocardiogr* 2010;23:1019-24.
20. Sengelov M, Jorgensen PG, Jensen JS, et al. Global Longitudinal Strain Is a Superior Predictor of All-Cause Mortality in Heart Failure With Reduced Ejection Fraction. *JACC Cardiovasc Imaging* 2015;8:1351-59.
21. Shah AM, Claggett B, Sweitzer NK, et al. Prognostic Importance of Impaired Systolic Function in Heart Failure With Preserved Ejection Fraction and the Impact of Spironolactone. *Circulation* 2015;132:402-14.
22. Hung CL, Verma A, Uno H, et al. Longitudinal and circumferential strain rate, left ventricular remodeling, and prognosis after myocardial infarction. *J Am Coll Cardiol* 2010;56:1812-22.
23. Ersboll M, Valeur N, Mogensen UM, et al. Prediction of all-cause mortality and heart failure admissions from global left ventricular longitudinal strain in patients with acute myocardial infarction and preserved left ventricular ejection fraction. *J Am Coll Cardiol* 2013;61:2365-73.
24. Bertini M, Ng AC, Antoni ML, et al. Global longitudinal strain predicts long-term survival in patients with chronic ischemic cardiomyopathy. *Circ Cardiovasc Imaging* 2012;5:383-91.
25. Thavendiranathan P, Poulin F, Lim KD, et al. Use of myocardial strain imaging by echocardiography for the early detection of cardiotoxicity in patients during and after cancer chemotherapy: a systematic review. *J Am Coll Cardiol* 2014;63:2751-68.
26. Yingchoncharoen T, Gibby C, Rodriguez LL, et al. Association of myocardial deformation with outcome in asymptomatic aortic stenosis with normal ejection fraction. *Circ Cardiovasc Imaging* 2012;5:719-25.
27. Liu H, Pozios I, Haileselassie B, et al. Role of Global Longitudinal Strain in Predicting Outcomes in Hypertrophic Cardiomyopathy. *Am J Cardiol* 2017;120:670-75.
28. Kuznetsova T, Herbots L, Richart T, et al. Left ventricular strain and strain rate in a general population. *Eur Heart J* 2008;29:2014-23.
29. Jensen MT, Sogaard P, Andersen HU, et al. Global longitudinal strain is not impaired in type 1 diabetes patients without albuminuria: the Thousand & 1 study. *JACC Cardiovasc Imaging* 2015;8:400-10.
30. Narayanan A, Aurigemma GP, Chinali M, et al. Cardiac mechanics in mild hypertensive heart disease: a speckle-strain imaging study. *Circ Cardiovasc Imaging* 2009;2:382-90.
31. Wong CY, O'Moore-Sullivan T, Leano R, et al. Alterations of left ventricular myocardial characteristics associated with obesity. *Circulation* 2004;110:3081-7.

For peer review only

For peer review only - http://bmjopen.bmj.com/site/about/guidelines.xhtml

32. Ma C, Chen J, Yang J, et al. Quantitative assessment of left ventricular function by 3-dimensional speckle-tracking echocardiography in patients with chronic heart failure: a meta-analysis. *J Ultrasound Med* 2014;33:287-95.
33. Morris DA, Ma XX, Belyavskiy E, et al. Left ventricular longitudinal systolic function analysed by 2D speckle-tracking echocardiography in heart failure with preserved ejection fraction: a meta-analysis. *Open Heart* 2017;4:e000630.
34. Shetye A, Nazir SA, Squire IB, et al. Global myocardial strain assessment by different imaging modalities to predict outcomes after ST-elevation myocardial infarction: A systematic review. *World J Cardiol* 2015;7:948-60.
35. Kalam K, Otahal P, Marwick TH. Prognostic implications of global LV dysfunction: a systematic review and meta-analysis of global longitudinal strain and ejection fraction. *Heart* 2014;100:1673-80.
36. Stanton T, Leano R, Marwick TH. Prediction of all-cause mortality from global longitudinal speckle strain: comparison with ejection fraction and wall motion scoring. *Circ Cardiovasc Imaging* 2009;2:356-64.
37. Moher D, Liberati A, Tetzlaff J, et al. Preferred reporting items for systematic reviews and meta-analyses: the PRISMA statement. *BMJ* 2009;339:b2535.
38. Shamseer L, Moher D, Clarke M, et al. Preferred reporting items for systematic review and meta-analysis protocols (PRISMA-P) 2015: elaboration and explanation. *BMJ* 2015;350:g7647.
39. Wells G, Shea B, O'Connell D, et al. Newcastle-Ottawa Quality Assessment Scale, Cohort Studies, 2014.
40. Sanderson S, Tatt ID, Higgins JP. Tools for assessing quality and susceptibility to bias in observational studies in epidemiology: a systematic review and annotated bibliography. *Int J Epidemiol* 2007;36:666-76.
41. Higgins JP, Thompson SG, Deeks JJ, et al. Measuring inconsistency in meta-analyses. *BMJ* 2003;327:557-60.
42. Higgins JP, Thompson SG. Quantifying heterogeneity in a meta-analysis. *Stat Med* 2002;21:1539-58.
43. Cochran WG. The comparison of percentages in matched samples. *Biometrika* 1950;37:256-66.
44. Sterne JA, Sutton AJ, Ioannidis JP, et al. Recommendations for examining and interpreting funnel plot asymmetry in meta-analyses of randomised controlled trials. *BMJ* 2011;343:d4002.

Figure legends

Figure 1 Flow diagram illustrates the study selection process following the PRISMA statement.[37]

For peer review only - http://bmjopen.bmj.com/site/about/guidelines.xhtml

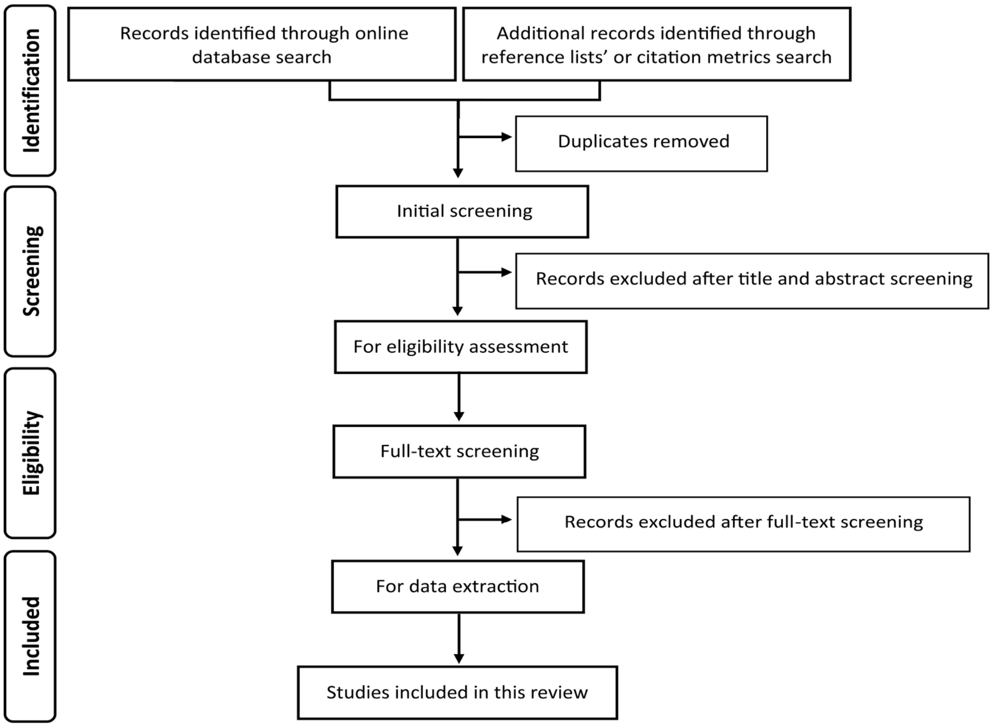

Figure 1 Flow diagram illustrates the study selection process following the PRISMA statement.37

99x73mm (600 x 600 DPI)

Supplementary file 1. Databases' search strategy

MEDLINE

Database: Ovid MEDLINE(R) Epub Ahead of Print, In-Process & Other Non-Indexed Citations, Ovid MEDLINE(R) Daily and Ovid MEDLINE(R) <1946 to Present>

Search Strategy:

----

1 Ventricular Function, Left/ (32622)
2 Ventricular Dysfunction, Left/ (24705)
3 left ventric*.mp. (179585)
4 LV.mp. (41504)
5 1 or 2 or 3 or 4 (198982)
6 Echocardiography/ (78179)
7 speckle tracking.mp. (3722)
8 STE.mp. (1905)
9 strain.mp. (385200)
10 deformation.mp. (36853)
11 mechanic*.mp. (390628)
12 torsion.mp. (22257)
13 twist.mp. (10298)
14 rotation.mp. (95760)
15 GLS.mp. (1508)
16 6 or 7 or 8 or 9 or 10 or 11 or 12 or 13 or 14 or 15 (963614)
17 Cardiovascular Diseases/ (128863)
18 cardiovascular disease*.mp. (220948)
19 Heart Failure/ (103286)
20 Heart failure.mp. (176882)
21 HF.mp. (35998)
22 Mortality/ (39168)
23 mortality.mp. (670200)
24 Death/ (16230)
25 death.mp. (691814)
26 Morbidity/ (27417)
27 morbidity.mp. (330859)
28 (cardi* adj3 (event* or outcome*)).mp. (66991)
29 17 or 18 or 19 or 20 or 21 or 22 or 23 or 24 or 25 or 26 or 27 or 28 (1690357)
30 predict*.mp. (1382430)
31 prognos*.mp. (734497)
32 30 or 31 (1934125)
33 exp Cohort Studies/ (1714168)
34 (cohort adj (study or studies)).mp. (310492)
35 (Follow up adj (study or studies)).mp. (604740)
36 Longitudinal.mp. (250750)
37 (observational adj (study or studies)).mp. (109821)
38 Epidemiologic studies/ (7611)

For peer review only

	39 (epidemiologic* adj (study or studies)).mp. (79971)
	40 population based study.mp. (24755)
	41 general population.mp. (85498)
	42 communit*.mp. (535197)
	43 33 or 34 or 35 or 36 or 37 or 38 or 39 or 40 or 41 or 42 (2539317)
	44 5 and 16 and 29 and 32 and 43 (2940)
**EMBASE**	Database: Embase Classic+Embase <1947 to 2018 February 27>
	Search Strategy:
	--
	1 heart left ventricle function/ (39176)
	2 left ventric*.mp. (332499)
	3 LV.mp. (83461)
	4 1 or 2 or 3 (353062)
	5 echocardiography/ (178295)
	6 exp speckle tracking echocardiography/ (3150)
	7 speckle tracking.mp. (10536)
	8 STE.mp. (4416)
	9 strain.mp. (762166)
	10 deformation.mp. (42935)
	11 mechanic*.mp. (498347)
	12 torsion.mp. (27446)
	13 twist.mp. (12502)
	14 rotation.mp. (109923)
	15 GLS.mp. (3938)
	16 5 or 6 or 7 or 8 or 9 or 10 or 11 or 12 or 13 or 14 or 15 (1553350)
	17 cardiovascular disease/ (241943)
	18 cardiovascular disease*.mp. (336057)
	19 heart failure/ (212076)
	20 Heart Failure.mp. (347509)
	21 HF.mp. (60866)
	22 mortality/ (722163)
	23 cardiovascular mortality/ (29486)
	24 all cause mortality/ (4807)
	25 mortality.mp. (1271143)
	26 death/ (284488)
	27 death.mp. (1097060)
	28 morbidity/ (309365)
	29 morbidity.mp. (569914)
	30 (cardi* adj3 (event* or outcome*)).mp. (110928)
	31 17 or 18 or 19 or 20 or 21 or 22 or 23 or 24 or 25 or 26 or 27 or 28 or 29 or 30 (2824491)
	32 predict*.mp. (1798936)
	33 prognos*.mp. (982782)

For peer review only

	34	32 or 33 (2544622)
	35	longitudinal study/ (109328)
	36	longitudinal.mp. (297876)
	37	prospective study/ (426930)
	38	cohort analysis/ (346132)
	39	(Cohort adj (study or studies)).mp. (217636)
	40	(follow up adj (study or studies)).mp. (63815)
	41	(observational adj (study or studies)).mp. (172984)
	42	(epidemiologic* adj (study or studies)).mp. (100634)
	43	population based study.mp. (33254)
	44	general population.mp. (124629)
	45	communit*.mp. (641283)
	46	35 or 36 or 37 or 38 or 39 or 40 or 41 or 42 or 43 or 44 or 45 (1963334)
	47	4 and 16 and 31 and 34 and 46 (4098)

'adj3' is a proximity operator and indicates within three words;

'OR and AND' are Boolean operators;

'*' indicates truncation;

'mp.' means a keyword search of title, abstract, original title, name of substance word, subject heading word and keyword heading word.

For peer review only

Eligibility Form: Prognostic Implications of Left Ventricular Strain by Speckle-Tracking Echocardiography in Population-based Studies

**1. Reference details**		
Ref ID		
Reference citation		
First author		
Year of publication		
Title of the paper		
Type of publication	Paper ☐	Abstract ☐
Assessor's identifier	1 ☐	2 ☐
Date		
Contact details of authors		

**2. Study eligibility**			
Inclusion of the study	Yes ☐ No ☐	Unclear ☐	
**Eligibility criteria**	**Reason for exclusion (if excluded)**	Yes	No
Longitudinal (cohort) study	• Ineligible study design e.g. Cross-sectional, case-control, or CRT	☐	☐
General population, community/population-based samples/ unselected community-dwelling individuals	• Ineligible population e.g. patients with established CVD, not population-based, unrepresentative samples of the general population	☐	☐
LV strain measured by STE	• Ineligible exposure e.g. measured by MRI	☐	☐
	• Ineligible outcome (Different from outcomes considered in this review e.g. LV remodelling)	☐	☐
	• Duplicate	☐	☐
	• Reviews, abstracts, letters, or conference proceeding	☐	☐
	• Other (please specify): e.g. not in English or in a peer reviewed journal	☐	☐
Notes:			

**Additional information**
Notes:

DO NOT PROCEED IF PAPER EXCLUDED FROM REVIEW

For peer review only

Data Extraction Form: Prognostic Implications of Left Ventricular Strain by Speckle-Tracking Echocardiography in Population-based Studies

**3. Study details**		
Ref ID		
Study (cohort) name		
Study design	Longitudinal (cohort) study ☐	
Region/country		
Sample size		
Mean (range) age	Mean ± SD :	Range (IQR):
Sex	Male, n (%):	Female, n (%):
Ethnicity		
Follow-up duration		
Clinical variables (n, %)		
• Hypertension		
• Diabetes mellitus		
• Dyslipidaemia		
• Smoking status		
• Known CVD		

For peer review only

**4. Exposure details**				
Hardware			Software	
Procedure:	2D-STE ☐	3D-STE ☐		
Measured LV strain (direction)			Images obtained from	number of LV segments
Longitudinal:	Yes ☐	No ☐		
Circumferential:	Yes ☐	No ☐		
Radial:	Yes ☐	No ☐		
Others (specify):	Yes ☐	No ☐		
Notes:				

**5. Outcomes' details**	
Type of outcomes used in this review	Outcomes reported in this study (n)
**Primary outcome(s)**<br>All-cause mortality	
**Secondary outcome (s)**<br>A. Composite **cardiovascular** (CV) end point includes: CV mortality, CHD events (MI, unstable angina, angina/ischemia requiring emergent hospitalization or revascularization), HF hospitalization, new	

onset AF, life threatening arrhythmia , recorded automatic implantable cardioverter defibrillator (AICD) shocks, stroke, transit ischemic attack, peripheral arterial disease with arterial revascularization procedure.	
B. Composite **cardiac** end point includes: CV mortality, CHD events, HF hospitalization, new onset AF, life threatening arrhythmia, recorded AICD shocks.	
Report an individual end point included in the composite cardiac or CV end point	Yes ☐ No ☐<br>List if yes:
Assessment of outcome	☐ independent blind assessment ☐ record linkage<br>☐ self-report ☐ others
Notes:	

**6. Available number of participants**		
		Number
Baseline sample size	Yes ☐ No ☐	
Excluded from the baseline	Yes ☐ No ☐<br>Reason if yes:	
Lost to follow-up	Yes ☐ No ☐	
Other losses	Yes ☐ No ☐	
Total number included in the analysis	Yes ☐ No ☐	
All subjects accounted for	Yes ☐ No ☐	
Notes:		

**7. Statistical analysis**	
Statistical method used	Cox proportional hazards regression Yes ☐ No ☐<br>Others if 'No':
Analysis includes males and females	Yes ☐ No ☐
Study reports results by sex	Yes ☐ No ☐
List all models used including unadjusted	

For peer review only

7. Results of the association of interest

Exposure	Outcome	Group (M/F/All)	Total (n) included in the analysis	Events/non-events	Effect estimate	95% CI, SE, p	Unit	Adjustments (none if unadjusted)

For peer review only

NEWCASTLE - OTTAWA QUALITY ASSESSMENT SCALE - COHORT STUDIES (Amended)

Note: A study can be awarded a maximum of one star for each numbered item within the Selection and Outcome categories.

Selection

1) Representativeness of the cohort
   a) Truly representative of the average person in the community ⋇
   b) Somewhat representative of the average person in the community (sampling described and designed to achieve adequately representative sample of the community)⋇
   c) Selected group e.g. nurses, volunteers
   d) No description of the derivation of the cohort
2) Assessment of exposure reliability
   a) Include assessment of inter- and intra-observer variabilities ⋇
   b) Include assessment of intra-observer variability ⋇
   c) No description
3) Completeness of collection of potential confounders1(i.e. CVD risk factors)
   a) Complete ⋇
   b) Partially complete (≥ 5) ⋇
   c) Partially complete (< 5)
   d) No description
4) Demonstration that the outcome of interest not present at start of study (and/or sensitively analysis)
   a) Yes ⋇
   b) No

Outcome

1) Assessment of outcome
   a) Independent blind assessment ⋇
   b) Record linkage ⋇
   c) Self-report
   d) No description
2) Was follow-up long enough for outcomes to occur
   a) yes (an adequate follow up period for outcome of interest is a year) ⋇
   b) no
3) Adequacy of follow up of cohorts
   a) Complete follow up - all subjects accounted for ⋇
   b) Subjects lost to follow up unlikely to introduce bias - small number lost - > 80% follow up, or description provided of those lost ⋇
   c) Follow up rate < 80% and no description of those lost
   d) No statement

The total quality score will be calculated by averaging the score of the two researchers with the range from 0 (lowest quality) to 7 (highest quality) and will be provided as a descriptive variable of the included studies.

---

1 Based on Framingham risk factors modified to include ethnicity: age, sex, blood pressure, antihypertensive medication (yes/no), total cholesterol, HDL cholesterol (or BMI), smoking, diabetes mellitus, and ethnicity.

For peer review only

Reporting checklist for protocol of a systematic review.

Based on the PRISMA-P guidelines.

Instructions to authors

Complete this checklist by entering the page numbers from your manuscript where readers will find each of the items listed below.

Your article may not currently address all the items on the checklist. Please modify your text to include the missing information. If you are certain that an item does not apply, please write "n/a" and provide a short explanation.

Upload your completed checklist as an extra file when you submit to a journal.

In your methods section, say that you used the PRISMA-P reporting guidelines, and cite them as:

Moher D, Shamseer L, Clarke M, Ghersi D, Liberati A, Petticrew M, Shekelle P, Stewart LA. Preferred Reporting Items for Systematic Review and Meta-Analysis Protocols (PRISMA-P) 2015 statement. Syst Rev. 2015;4(1):1.

		Reporting Item	Page Number
Identification	#1a	Identify the report as a protocol of a systematic review	1
Update	#1b	If the protocol is for an update of a previous systematic review, identify as such	n/a
	#2	If registered, provide the name of the registry (such as PROSPERO) and registration number	7
Contact	#3a	Provide name, institutional affiliation, e-mail address of all protocol authors; provide physical mailing address of corresponding author	1
Contribution	#3b	Describe contributions of protocol authors and identify the guarantor of the review	13
	#4	If the protocol represents an amendment of a previously completed or published protocol, identify as such and list changes; otherwise, state plan for documenting important protocol amendments	n/a

For peer review only

Sources	#5a	Indicate sources of financial or other support for the review	13
Sponsor	#5b	Provide name for the review funder and / or sponsor	n/a
Role of sponsor or funder	#5c	Describe roles of funder(s), sponsor(s), and / or institution(s), if any, in developing the protocol	n/a
Rationale	#6	Describe the rationale for the review in the context of what is already known	4, 5, 6
Objectives	#7	Provide an explicit statement of the question(s) the review will address with reference to participants, interventions, comparators, and outcomes (PICO)	5, 6
Eligibility criteria	#8	Specify the study characteristics (such as PICO, study design, setting, time frame) and report characteristics (such as years considered, language, publication status) to be used as criteria for eligibility for the review	7, 8, 9
Information sources	#9	Describe all intended information sources (such as electronic databases, contact with study authors, trial registers or other grey literature sources) with planned dates of coverage	9, 10
Search strategy	#10	Present draft of search strategy to be used for at least one electronic database, including planned limits, such that it could be repeated	9 (S file 1)
Study records - data management	#11a	Describe the mechanism(s) that will be used to manage records and data throughout the review	9
Study records - selection process	#11b	State the process that will be used for selecting studies (such as two independent reviewers) through each phase of the review (that is, screening, eligibility and inclusion in meta-analysis)	9, 10
Study records - data collection process	#11c	Describe planned method of extracting data from reports (such as piloting forms, done independently, in duplicate), any processes for obtaining and confirming data from investigators	10, 11
Data items	#12	List and define all variables for which data will be sought	10, 11

For peer review only

		(such as PICO items, funding sources), any pre-planned data assumptions and simplifications	
Outcomes and prioritization	#13	List and define all outcomes for which data will be sought, including prioritization of main and additional outcomes, with rationale	8
Risk of bias in individual studies	#14	Describe anticipated methods for assessing risk of bias of individual studies, including whether this will be done at the outcome or study level, or both; state how this information will be used in data synthesis	11, 12
Data synthesis	#15a	Describe criteria under which study data will be quantitatively synthesised	12
	#15b	If data are appropriate for quantitative synthesis, describe planned summary measures, methods of handling data and methods of combining data from studies, including any planned exploration of consistency (such as I2, Kendall's τ)	11, 12
	#15c	Describe any proposed additional analyses (such as sensitivity or subgroup analyses, meta-regression)	11, 12
	#15d	If quantitative synthesis is not appropriate, describe the type of summary planned	12
Meta-bias(es)	#16	Specify any planned assessment of meta-bias(es) (such as publication bias across studies, selective reporting within studies)	12
Confidence in cumulative evidence	#17	Describe how the strength of the body of evidence will be assessed (such as GRADE)	n/a (justification provided in p11)

The PRISMA-P checklist is distributed under the terms of the Creative Commons Attribution License CC-BY 4.0. This checklist can be completed online using https://www.goodreports.org/, a tool made by the EQUATOR Network in collaboration with Penelope.ai

For peer review only

BMJ Open

Prognostic Implications of Left Ventricular Strain by Speckle-Tracking Echocardiography in Population-based Studies: A Systematic Review Protocol of the Published Literature

Journal:	*BMJ Open*
Manuscript ID	bmjopen-2018-023346.R1
Article Type:	Protocol
Date Submitted by the Author:	23-May-2018
Complete List of Authors:	Al Saikhan, Lamia; University College London , Institute of Cardiovascular Science ; Imam Abdulrahman Bin Faisal University, Department of Cardiac Technology, College of Applied Medial Sciences<br>Park, Chloe; University College London , Institute of Cardiovascular Science<br>Hardy, Rebecca; University College London, MRC Unit for Lifelong Health and Ageing<br>Hughes, A; University College London, Institute of Cardiovascular Science
**Primary Subject Heading**:	Cardiovascular medicine
Secondary Subject Heading:	Epidemiology
Keywords:	Speckle tracking echocardiography, Community dwelling individuals, Left ventricular strain, Mortality, Cardiovascular disease

For peer review only

SCHOLARONE™
Manuscripts

Prognostic Implications of Left Ventricular Strain by Speckle-Tracking Echocardiography in Population-based Studies: A Systematic Review Protocol of the Published Literature

Lamia Al Saikhan[1, 2], Chloe Park[1], Rebecca Hardy[3], Alun Hughes[1]

[1]Institute of Cardiovascular Science, School of Life and Medical Sciences, University College London, London, UK

[2]Department of Cardiac Technology, College of Applied Medial Sciences, Imam Abdulrahman Bin Faisal University, Dammam, Kingdom of Saudi Arabia

[3]MRC Unit for Lifelong Health and Ageing, University College London, London, UK.

lamia.saikhan.16@ucl.ac.uk

chloe.park@ucl.ac.uk

rebecca.hardy@ucl.ac.uk

alun.hughes@ucl.ac.uk

Correspondence:

Lamia Khaled Al Saikhan

Institute of Cardiovascular Science

University College London

Gower Street

London WC1E 6BT

lamia.saikhan.16@ucl.ac.uk

Tel: 020 7677 1857

Word count: 2157 (excluding title page, abstract, references, figures and tables)

Keywords: Community dwelling individuals, Speckle tracking echocardiography, Mortality, Cardiovascular disease, Left ventricular strain.

For peer review only - http://bmjopen.bmj.com/site/about/guidelines.xhtml

ABSTRACT

Introduction: Left ventricular (LV) strain by speckle-tracking echocardiography (STE) is a comparatively new prognostic marker. Meta-analyses relating LV strain by STE to outcomes have been conducted in selected patient-based populations with established or suspected cardiovascular (CV) diseases. However, the evidence related to population-based studies of community dwelling individuals is uncertain. The aim of this study is to provide a comprehensive systematic review and analysis of the current available literature regarding LV strain by STE as a predictor of adverse outcomes in population-based studies.

Methods and analyses: Thesaurus and text-word searching will be used to search two online databases (MEDLINE and EMBASE) and additional sources will be identified from citation metrics and reference lists' search. Dual search results' screening, data extraction and quality assessment will be performed. Cohort studies of community/population-based samples who have had STE and followed-up longitudinally for mortal and morbid events, and published in English and peer reviewed journals will be included. Primary outcome will be all-cause mortality whereas secondary outcomes will be composite cardiac and CV end points. Risk of bias will be assessed using Newcastle-Ottawa quality assessment scale of cohort studies that will be modified as appropriate. Any arising discrepancies will be discussed and resolved through consensus.

Ethics and dissemination: Ethical approval is not required as this is a protocol for a systematic review. The findings of this study will be presented at scientific conferences and published in a peer reviewed journal. Any amendments to the protocol will be documented and updated in the PROSPERO registry.

Systematic review registration number: CRD42018090302.

For peer review only

For peer review only - http://bmjopen.bmj.com/site/about/guidelines.xhtml

ARTICLE SUMMARY

Strengths and limitations of this study

- This systematic review will evaluate the evidence related to the incremental prognostic value of left ventricular (LV) strain by speckle tracking echocardiography (STE) in relation to mortality and cardiovascular events in community dwelling individuals or population-based samples known to be at low risk relative to selected diseased populations.
- The results of this systematic review will add to the existing evidence on the utility of STE-based LV strain as a measure of cardiac function and risk, and influence its use in longitudinal population-based studies.
- Because of the anticipated heterogeneity in results/presentation of results and/or study design across a limited number of existing studies, there may be a limited scope for meta-analysis. When meta-analysis is possible, heterogeneity in associations between studies may be difficult to explain.

For peer review only

For peer review only - http://bmjopen.bmj.com/site/about/guidelines.xhtml

INTRODUCTION

Cardiovascular disease (CVD) is the leading cause of mortality worldwide and accounts for 31% of deaths.[1] Assessment of left ventricular (LV) global systolic function is central in evaluating prognosis, as well as in determining therapeutic strategies in CVD.[2] LV ejection fraction (LVEF) is a simple and widely used parameter that is most commonly measured by echocardiography.[3] Reduced LVEF is known to be associated with unfavorable outcomes in populations with established CVD,[4-7] but also in people without known CVD.[8 9] However, this parameter suffers from a number of inherent limitations including load dependency, low reproducibility, and geometric assumptions.[3] Furthermore, its inverse association with outcomes varies considerably across the spectrum of LVEF, being greatest in moderately to severely impaired LV systolic function.[7]

LV strain imaging, tissue Doppler- and speckle tracking- based, has emerged as a powerful and sensitive tool allowing an accurate quantification of myocardial mechanics including longitudinal, circumferential and radial shortening/lengthening, and torsion.[10] Strain, a dimensionless index, is defined as a change in length of a myocardial segment: strain (ε) $=\Delta L/L_0$, where ΔL=change in length and L_0= original length.[3 10 11] The major limitation of tissue Doppler-derived strain (natural strain) is the angle dependency of measurements and this technique has been superseded by speckle-tracking echocardiography [STE] (Lagrangian strain).[10] As the term implies, STE is based on the analysis of myocardial features (speckles) on grayscale B-mode images that are generated by ultrasound interference patterns within the myocardium.[10-12] These speckles are tracked frame by frame during the cardiac cycle providing a sensitive and objective measure of global and regional myocardial function (i.e. Lagrangian strain).[10-12] Unlike tissue Doppler imaging (TDI), STE is angle independent, can differentiate active from passive motion,[13] and has been validated in an

For peer review only - http://bmjopen.bmj.com/site/about/guidelines.xhtml

experimental setting against sonomicrometry[14] as well as in a clinical setting against cardiac magnetic resonance imaging (CMR).[15]

STE-based LV strain imaging has been shown to have an independent and additive prognostic value over conventional echocardiographic measures in a number of studies of diseased populations. These included patients with chronic[16 17] or acute[18] heart failure (HF) including both people with reduced[19 20] and preserved[21] LVEF. Further, STE-based LV strain imaging has prognostic utility after acute myocardial infarction (MI)[22 23] and in chronic ischemic cardiomyopathy.[24] Importantly, STE-based LV strain especially global longitudinal strain (GLS) has a potential value in detecting subclinical left ventricular systolic dysfunction (LVSD) when LVEF is within normal limits.[3] Indeed, the most common clinical setting is the prediction of cardiotoxicity in patients receiving cancer thereby.[25] GLS has also shown an association with unfavorable outcomes in asymptomatic severe aortic stenosis[26] and hypertrophic cardiomyopathy[27] with preserved LVEF. Alterations in GLS despite preserved LVEF have been demonstrated in populations with risk factors that predispose to CVD including aging[28], diabetes mellitus (DM)[29], hypertension[30] and obesity[31], and may be the earliest marker of LVSD.

Previous systematic reviews and meta-analyses relating LV strain by STE to outcomes have been conducted in selected patient-based samples with established[32-35] or established plus suspected CVD.[36] However, no systematic review has been performed of studies which have recruited community dwelling individuals or population-based samples, who were not selected on the basis of disease or clinical status, and are known to be at low risk compared to selected diseased populations, and the utility of STE in this setting is uncertain. This may be important since selecting samples based on disease status can introduce bias.[37] Consequently, we sought to investigate whether LV strain measured by STE is associated with risk of total and cardiovascular (CV) mortality and morbidity

For peer review only - http://bmjopen.bmj.com/site/about/guidelines.xhtml

independent of conventional risk factors in population-based samples. We therefore proposed to carry out a systematic review and, where possible, meta-analysis of the current literature relating LV strain by STE to mortality and CV morbidity in the general population.

For peer review only

For peer review only - http://bmjopen.bmj.com/site/about/guidelines.xhtml

MEFTHODS

Protocol registration

The protocol of this review is registered with the PROSPERO database (systematic review registration number: CRD42018090302).

Reporting

This systematic review will be conducted and reported in adherence with Preferred Reporting Items for Systematic Reviews and Meta-Analysis (PRISMA) statement[38] and PRISMA-protocols.[39]

Eligibility criteria

Inclusion criteria

The inclusion criteria which relate to the type of study design, population, and measurement procedure are summarized in table 1. Studies will only be included if they assess the prospective association of LV strain with at least one of the pre-specified outcomes in community-dwelling individuals who were not selected on the basis of disease or clinical status_(table 1). To be eligible for inclusion, studies will be required to include a statement regarding the ethical approval, or adherence to an appropriate standard (such as the declaration of Helsinki).[40]

For peer review only - http://bmjopen.bmj.com/site/about/guidelines.xhtml

Table 1 Eligibility criteria of the systematic review	
Type of Study design	- longitudinal (cohort) studies, including placebo limbs of population-based clinical trials
Type of participants/population	- Adults (>18 years)<br>- General population, community/population-based samples, or community-dwelling individuals not selected on the basis of disease or clinical status
Type of procedure	- Speckle tracking echocardiography (STE)
Measured parameters (exposure)	- LV strain measured at rest by STE in any direction of the myocardial motion (i.e. global longitudinal strain and/or circumferential strain and/or radial strain and/or transverse strain, and/or any STE derived parameters, e.g. torsion and/or twist)
Type of outcomes	- Primary outcome: All-cause mortality<br>- Secondary outcomes: Composite cardiovascular and cardiac end points (see text for details)
Year of publication	- No limit applied
Languages	- Articles published in English
Publication status	- Published in a peer-reviewed journal

Outcomes

The primary outcome will be all-cause mortality. Secondary outcomes will be (a) composite CV end points, including any combination of CV mortality, coronary heart disease (CHD) events (MI, unstable angina, angina/ischemia requiring emergent hospitalization or revascularization), HF hospitalization, new onset atrial fibrillation (AF), life threatening arrhythmia, recorded automatic implantable cardioverter defibrillator (AICD) shocks, stroke, transit ischemic attack, or peripheral arterial disease with arterial revascularization procedure, or (b) composite cardiac end point, including any combination of CV mortality, CHD events (MI, unstable angina, angina/ischemia requiring emergent hospitalization, or

For peer review only

For peer review only - http://bmjopen.bmj.com/site/about/guidelines.xhtml

revascularization), HF hospitalization, new onset AF, life threatening arrhythmia, recorded AICD shocks. Tertiary outcomes will be any individual secondary end point included in the composite cardiac or CV end point.

Exclusion criteria

The exclusion criteria will be as follows: (1) studies not meeting the inclusion criteria; (2) case-control studies, cross-sectional studies, or randomized controlled trials which lack a placebo limb; (3) studies in unrepresentative samples of the general population (e.g. clinic populations) or patients with established/known CVD (e.g. patients with MI or HF); (4) LV strain measured by TDI or by another imaging modality (e.g. CMR); (5) studies with end points that do not match those specified in this review (e.g. LV remodeling); or (6) abstracts, reviews, conference preceding or letters to the editorial.

Search Strategy

Electronic searches

This literature search will be performed systematically in the following online databases: MEDLINE (1946 to present) and EMBASE Classic + EMBASE (1947 to present) via OvidSP interface**.** Research has suggested that these databases are likely to be sufficient to identify relevant studies.[41] A combination of thesaurus and text-word searching will be used to comprehensively extract all relevant articles. Boolean operators (AND/OR), proximity operators and truncation commands will be used when necessary to narrow the search to the relevant literature. The effectiveness of the search stagey will be tested and refined accordingly. The relevant thesaurus terms will be developed first for the MEDLINE database and will be checked and modified for EMBASE database as appropriate. A modified version of SIGN filters for observational studies will be used to capture the required studies (i.e.

For peer review only - http://bmjopen.bmj.com/site/about/guidelines.xhtml

population –based studies only). No time or language limit will be applied to the search and the final date on which the search will be carried out will be stated. Databases' search strategy is shown in online supplementary file 1.

Additional sources

Other sources will be identified by thoroughly searching the reference lists of all relevant articles. We will also search the citation metrics of those articles using Web of Science Core Collection for additional studies.

Patient and Public Involvement

Patients and the public were not involved in designing the protocol of this systematic review.

DATA COLLECTION AND ANALYSES

Study selection

The search results from each database will be retrieved and merged using reference manger software (i.e. in a single EndNote library). Duplicates will be identified using the command 'Find Duplicates' and will be removed before screening is commenced. Titles and abstracts will then be screened for eligibility by two researchers working independently and irrelevant articles will be dismissed. Full-texts of potentially eligible articles will be retrieved and double screened. A predefined eligibility form (see supplementary file 2) will be applied to the identified studies to determine whether the inclusion criteria are met. Reasons for exclusions will be recorded. Discrepancies will be reviewed and resolved through consensus of all reviewers. The study selection process will be summarized using PRISMA flow diagram (figure 1).

For peer review only - http://bmjopen.bmj.com/site/about/guidelines.xhtml

Data extraction

We will use a predefined data extraction form (see supplementary file 2) which will be piloted on a sample of included studies to ensure that all relevant information is captured. Data will be extracted by two researchers independent of each other and any discrepancies will be discussed in order to achieve consensus. The following data will be extracted: (1) citation details including title, type and year of publication; (2) study and participant details including name, region and design of the study, sample size, demographics (age, sex ethnicity), and follow up duration; (3) clinical variables including the presence or absence of hypertension, DM, dyslipidemia, smoking status and known CVD; (4) exposure details including the hardware and the software used for the acquisition and the analysis as well as details regarding the measured LV strain including the direction of the analyzed myocardial motion, the echocardiographic images used for the analysis, the number of LV segments involved, the number of sonographers used to perform the analysis, and if intra- or inter-observer variability was reported; (5) details of outcomes (i.e. all-cause mortality, composite CV and cardiac end points) including how these were ascertained; (6) statistical methods used e.g. Cox proportional hazards regression, as well as statistics related to the association of interest e.g. hazard (or rate) ratio, related measures of precision (i.e. 95% confidence interval or standard error) and statistical significance (P-value); and (7) information related to adjustment for potential confounders.

Quality assessment

Quality of included studies will be assessed using a pre-assigned scale (Newcastle-Ottawa quality assessment scale of cohort studies)[42] that will be modified according to the review question to capture all relevant sources of potential bias (see supplementary file 3).[43] Two reviewers will judge the quality of each study by considering the following criteria:

For peer review only - http://bmjopen.bmj.com/site/about/guidelines.xhtml

Representativeness of the cohort, completeness of collection of potential confounders, follow-up duration, assessment of outcome and the reliability of exposure (i.e. LV strain). The total quality score will be reported as the average of the two researchers' scores ranging from 0, the lowest quality score, to 7, the highest quality score. Studies will be included irrespective of the quality assessment, but sensitivity analyses will be carried out to test the impact of removing the studies with the lowest quality score one at a time.

This systematic review does not study a treatment comparison, and hence quality of evidence assessment based on strict GRADE guidelines (i.e. Grading Quality of Evidence and Strength of Recommendations) is not directly applicable. However, we will employ relevant components of a GRADE evaluation, namely quality of study, consistency and effect size in evaluating the strength of evidence; the issue of generalizability is dealt with through the inclusion/exclusion criteria.

Data synthesis

Descriptive summary of the characteristics and findings of the included studies will be provided in tables. We anticipate that there may be a limited scope for meta-analysis due to differences in the method of analysis and the reporting of results across studies. If the included studies are sufficiently homogenous and report the same statistics (e.g. comparable hazard ratios), a quantitative synthesis using random effect meta-analysis will be used to pool the results of the association of interest. Based on our expectation, we will likely to use the hazard ratio and 95% confidence interval as an effect estimate for presenting the results, and will present the data graphically in forest plots.

The degree of heterogeneity will be assessed using Higgins Thompson I^2 test[44 45] and Cochran's Q test.[46] If data allow, we will perform subgroup analyses or meta-regression to explore pre-specified potential sources of between-study heterogeneity: a) study quality, b)

For peer review only - http://bmjopen.bmj.com/site/about/guidelines.xhtml

variation in exposure measurement (e.g. vendors and type of strain [i.e. longitudinal, circumferential or radial]), and c) variation in CV and cardiac outcomes definition. Although it may not be possible, we plan to do subgroup analyses by sex and age categories (<65 and ≥65 years). Possible publication bias will be assessed using a Funnel plot.[47]

For peer review only

For peer review only - http://bmjopen.bmj.com/site/about/guidelines.xhtml

DISCUSSION

STE is increasingly and widely used in clinical practice. However, this technology suffers from inherent limitations, being influenced by technical as well as clinical factors.[13] High quality images with adequate frame rates are required and there is limited evidence comparing different imaging modalities (2D and 3D echocardiography). Further, this technology is vendor specific: differences between vendors in data processing and analysis limit its generalizability, and contribute to the present lack of normal values.[13 48] STE-derived strain values may also be influenced by clinical factors including age, sex and race.[13 48]

This systematic review will identify and synthesize evidence regarding STE-derived measures as prognostic indicators of mortality and CV events in population-based/community dwelling individuals. The restriction to studies not selected on the basis of disease or clinical status should minimize potential collider bias due to index case selection. The review will also potentially highlight known limitations of STE. This analysis will add to evidence on the utility of STE as a measure of cardiac function and risk, and influence its use (or otherwise) in longitudinal population-based studies and community dwelling samples.

ETHICS AND DISSEMINATION:

This protocol is for conducting a systematic review and hence no ethical approval is required. The findings of this review will be presented at scientific conferences and published in a peer-reviewed journal.

Author Statement /Contributors

LA is the guarantor of the review who drafted the protocol and registered it in PROSPERO. LA and AH contributed in developing the eligibility criteria, search strategy, and data extraction and quality assessment strategy. AH and CP critically reviewed and amended the

For peer review only - http://bmjopen.bmj.com/site/about/guidelines.xhtml

protocol. RH provided statistical advice and critically reviewed and commented on the protocol.

Funding statement None

Competing interest statement None declared

Provenance and peer review Not commissioned; externally peer reviewed.

Acknowledgments

AH works in a unit that receives support from the UK Medical Research Council (Programme Code MC_UU_12019/1) and also receives support from the British Heart Foundation (PG/15/75/31748, CS/15/6/31468, CS/13/1/30327), and the National Institute for Health Research University College London Hospitals Biomedical Research Centre. CP receives support from the British Heart Foundation (CS/15/6/31468). LA is supported by a scholarship grant from Imam Abdulrahman Bin Faisal University.

REFERNCES

1. WHO. Cardiovascular diseases (CVDs) [Internet]. WHO [updated May 2017. Available from: http://www.who.int/mediacentre/factsheets/fs317/en/. accessed 12 March 2018.
2. Ponikowski P, Voors AA, Anker SD, et al. 2016 ESC Guidelines for the diagnosis and treatment of acute and chronic heart failure: The Task Force for the diagnosis and treatment of acute and chronic heart failure of the European Society of Cardiology (ESC)Developed with the special contribution of the Heart Failure Association (HFA) of the ESC. *Eur Heart J* 2016;37:2129-200.
3. Potter E, Marwick TH. Assessment of Left Ventricular Function by Echocardiography: The Case for Routinely Adding Global Longitudinal Strain to Ejection Fraction. *JACC Cardiovasc Imaging* 2018;11:260-74.
4. Cohn JN, Johnson GR, Shabetai R, et al. Ejection fraction, peak exercise oxygen consumption, cardiothoracic ratio, ventricular arrhythmias, and plasma norepinephrine as determinants of prognosis in heart failure. The V-HeFT VA Cooperative Studies Group. *Circulation* 1993;87:VI5-16.
5. Gomes JA, Mehta D, Ip J, et al. Predictors of long-term survival in patients with malignant ventricular arrhythmias. *Am J Cardiol* 1997;79:1054-60.

For peer review only - http://bmjopen.bmj.com/site/about/guidelines.xhtml

6. Gottdiener JS, McClelland RL, Marshall R, et al. Outcome of congestive heart failure in elderly persons: influence of left ventricular systolic function. The Cardiovascular Health Study. *Ann Intern Med* 2002;137:631-9.
7. Curtis JP, Sokol SI, Wang Y, et al. The association of left ventricular ejection fraction, mortality, and cause of death in stable outpatients with heart failure. *J Am Coll Cardiol* 2003;42:736-42.
8. Hays AG, Sacco RL, Rundek T, et al. Left ventricular systolic dysfunction and the risk of ischemic stroke in a multiethnic population. *Stroke* 2006;37:1715-9.
9. Yeboah J, Rodriguez CJ, Stacey B, et al. Prognosis of individuals with asymptomatic left ventricular systolic dysfunction in the multi-ethnic study of atherosclerosis (MESA). *Circulation* 2012;126:2713-9.
10. Mor-Avi V, Lang RM, Badano LP, et al. Current and evolving echocardiographic techniques for the quantitative evaluation of cardiac mechanics: ASE/EAE consensus statement on methodology and indications endorsed by the Japanese Society of Echocardiography. *J Am Soc Echocardiogr* 2011;24:277-313.
11. Nesbitt GC, Mankad S, Oh JK. Strain imaging in echocardiography: methods and clinical applications. *Int J Cardiovasc Imaging* 2009;25 Suppl 1:9-22.
12. Mondillo S, Galderisi M, Mele D, et al. Speckle-tracking echocardiography: a new technique for assessing myocardial function. *Journal of ultrasound in medicine : official journal of the American Institute of Ultrasound in Medicine* 2011;30:71-83.
13. Collier P, Phelan D, Klein A. A Test in Context: Myocardial Strain Measured by Speckle-Tracking Echocardiography. *J Am Coll Cardiol* 2017;69:1043-56.
14. Langeland S, D'Hooge J, Wouters PF, et al. Experimental validation of a new ultrasound method for the simultaneous assessment of radial and longitudinal myocardial deformation independent of insonation angle. *Circulation* 2005;112:2157-62.
15. Amundsen BH, Helle-Valle T, Edvardsen T, et al. Noninvasive myocardial strain measurement by speckle tracking echocardiography: validation against sonomicrometry and tagged magnetic resonance imaging. *J Am Coll Cardiol* 2006;47:789-93.
16. Nahum J, Bensaid A, Dussault C, et al. Impact of longitudinal myocardial deformation on the prognosis of chronic heart failure patients. *Circ Cardiovasc Imaging* 2010;3:249-56.
17. Zhang KW, French B, May Khan A, et al. Strain improves risk prediction beyond ejection fraction in chronic systolic heart failure. *J Am Heart Assoc* 2014;3:e000550.
18. Cho GY, Marwick TH, Kim HS, et al. Global 2-dimensional strain as a new prognosticator in patients with heart failure. *J Am Coll Cardiol* 2009;54:618-24.
19. Mignot A, Donal E, Zaroui A, et al. Global longitudinal strain as a major predictor of cardiac events in patients with depressed left ventricular function: a multicenter study. *J Am Soc Echocardiogr* 2010;23:1019-24.
20. Sengelov M, Jorgensen PG, Jensen JS, et al. Global Longitudinal Strain Is a Superior Predictor of All-Cause Mortality in Heart Failure With Reduced Ejection Fraction. *JACC Cardiovasc Imaging* 2015;8:1351-59.
21. Shah AM, Claggett B, Sweitzer NK, et al. Prognostic Importance of Impaired Systolic Function in Heart Failure With Preserved Ejection Fraction and the Impact of Spironolactone. *Circulation* 2015;132:402-14.

For peer review only - http://bmjopen.bmj.com/site/about/guidelines.xhtml

22. Hung CL, Verma A, Uno H, et al. Longitudinal and circumferential strain rate, left ventricular remodeling, and prognosis after myocardial infarction. *J Am Coll Cardiol* 2010;56:1812-22.
23. Ersboll M, Valeur N, Mogensen UM, et al. Prediction of all-cause mortality and heart failure admissions from global left ventricular longitudinal strain in patients with acute myocardial infarction and preserved left ventricular ejection fraction. *J Am Coll Cardiol* 2013;61:2365-73.
24. Bertini M, Ng AC, Antoni ML, et al. Global longitudinal strain predicts long-term survival in patients with chronic ischemic cardiomyopathy. *Circ Cardiovasc Imaging* 2012;5:383-91.
25. Thavendiranathan P, Poulin F, Lim KD, et al. Use of myocardial strain imaging by echocardiography for the early detection of cardiotoxicity in patients during and after cancer chemotherapy: a systematic review. *J Am Coll Cardiol* 2014;63:2751-68.
26. Yingchoncharoen T, Gibby C, Rodriguez LL, et al. Association of myocardial deformation with outcome in asymptomatic aortic stenosis with normal ejection fraction. *Circ Cardiovasc Imaging* 2012;5:719-25.
27. Liu H, Pozios I, Haileselassie B, et al. Role of Global Longitudinal Strain in Predicting Outcomes in Hypertrophic Cardiomyopathy. *Am J Cardiol* 2017;120:670-75.
28. Kuznetsova T, Herbots L, Richart T, et al. Left ventricular strain and strain rate in a general population. *Eur Heart J* 2008;29:2014-23.
29. Jensen MT, Sogaard P, Andersen HU, et al. Global longitudinal strain is not impaired in type 1 diabetes patients without albuminuria: the Thousand & 1 study. *JACC Cardiovasc Imaging* 2015;8:400-10.
30. Narayanan A, Aurigemma GP, Chinali M, et al. Cardiac mechanics in mild hypertensive heart disease: a speckle-strain imaging study. *Circ Cardiovasc Imaging* 2009;2:382-90.
31. Wong CY, O'Moore-Sullivan T, Leano R, et al. Alterations of left ventricular myocardial characteristics associated with obesity. *Circulation* 2004;110:3081-7.
32. Ma C, Chen J, Yang J, et al. Quantitative assessment of left ventricular function by 3-dimensional speckle-tracking echocardiography in patients with chronic heart failure: a meta-analysis. *J Ultrasound Med* 2014;33:287-95.
33. Morris DA, Ma XX, Belyavskiy E, et al. Left ventricular longitudinal systolic function analysed by 2D speckle-tracking echocardiography in heart failure with preserved ejection fraction: a meta-analysis. *Open Heart* 2017;4:e000630.
34. Shetye A, Nazir SA, Squire IB, et al. Global myocardial strain assessment by different imaging modalities to predict outcomes after ST-elevation myocardial infarction: A systematic review. *World J Cardiol* 2015;7:948-60.
35. Kalam K, Otahal P, Marwick TH. Prognostic implications of global LV dysfunction: a systematic review and meta-analysis of global longitudinal strain and ejection fraction. *Heart* 2014;100:1673-80.
36. Stanton T, Leano R, Marwick TH. Prediction of all-cause mortality from global longitudinal speckle strain: comparison with ejection fraction and wall motion scoring. *Circ Cardiovasc Imaging* 2009;2:356-64.
37. Flanders WD, Eldridge RC, McClellan W. A nearly unavoidable mechanism for collider bias with index-event studies. *Epidemiology (Cambridge, Mass)* 2014;25:762-4.

For peer review only - http://bmjopen.bmj.com/site/about/guidelines.xhtml

38. Moher D, Liberati A, Tetzlaff J, et al. Preferred reporting items for systematic reviews and meta-analyses: the PRISMA statement. *BMJ* 2009;339:b2535.
39. Shamseer L, Moher D, Clarke M, et al. Preferred reporting items for systematic review and meta-analysis protocols (PRISMA-P) 2015: elaboration and explanation. *BMJ* 2015;350:g7647.
40. Vergnes JN, Marchal-Sixou C, Nabet C, et al. Ethics in systematic reviews. *J Med Ethics* 2010;36:771-4.
41. Hartling L, Featherstone R, Nuspl M, et al. The contribution of databases to the results of systematic reviews: a cross-sectional study. *BMC Medical Research Methodology* 2016;16:127.
42. Wells G, Shea B, O'Connell D, et al. Newcastle-Ottawa Quality Assessment Scale, Cohort Studies, 2014.
43. Sanderson S, Tatt ID, Higgins JP. Tools for assessing quality and susceptibility to bias in observational studies in epidemiology: a systematic review and annotated bibliography. *Int J Epidemiol* 2007;36:666-76.
44. Higgins JP, Thompson SG, Deeks JJ, et al. Measuring inconsistency in meta-analyses. *BMJ* 2003;327:557-60.
45. Higgins JP, Thompson SG. Quantifying heterogeneity in a meta-analysis. *Stat Med* 2002;21:1539-58.
46. Cochran WG. The comparison of percentages in matched samples. *Biometrika* 1950;37:256-66.
47. Sterne JA, Sutton AJ, Ioannidis JP, et al. Recommendations for examining and interpreting funnel plot asymmetry in meta-analyses of randomised controlled trials. *BMJ* 2011;343:d4002.
48. Yingchoncharoen T, Agarwal S, Popovic ZB, et al. Normal ranges of left ventricular strain: a meta-analysis. *J Am Soc Echocardiogr* 2013;26:185-91.

Figure legends

Figure 1 Flow diagram illustrates the study selection process following the PRISMA statement.[38]

For peer review only

For peer review only - http://bmjopen.bmj.com/site/about/guidelines.xhtml

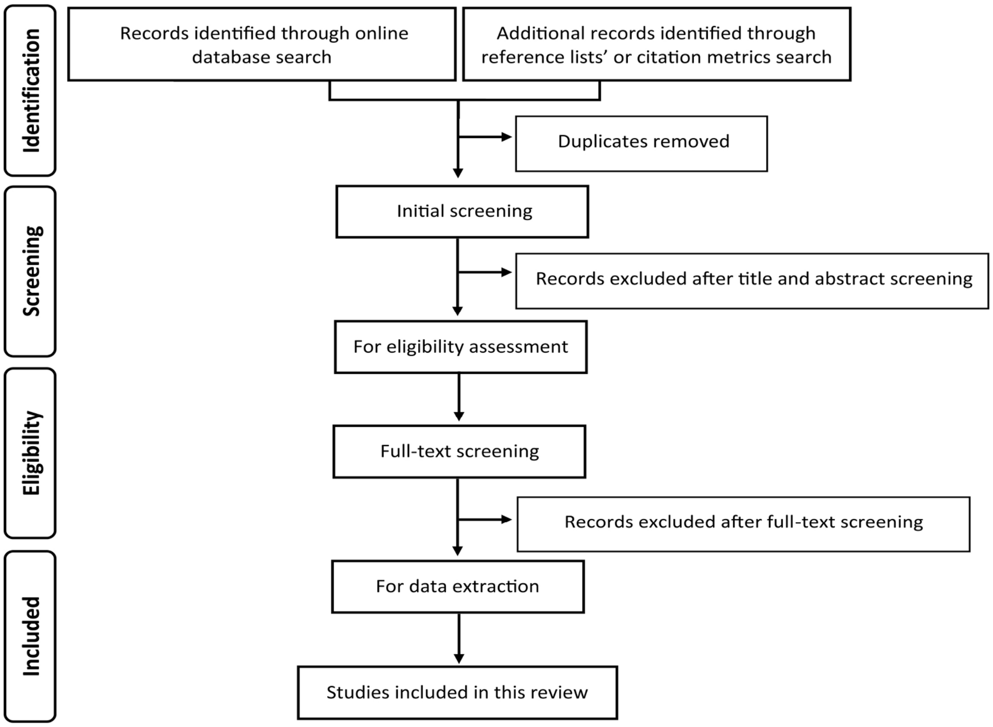

Figure 1 Flow diagram illustrates the study selection process following the PRISMA statement.37

99x73mm (600 x 600 DPI)

Supplementary file 1. Databases' search strategy

**MEDLINE**	Database: Ovid MEDLINE(R) Epub Ahead of Print, In-Process & Other Non-Indexed Citations, Ovid MEDLINE(R) Daily and Ovid MEDLINE(R) <1946 to Present><br>Search Strategy:<br>--<br>1 Ventricular Function, Left/ (32622)<br>2 Ventricular Dysfunction, Left/ (24705)<br>3 left ventric*.mp. (179585)<br>4 LV.mp. (41504)<br>5 1 or 2 or 3 or 4 (198982)<br>6 Echocardiography/ (78179)<br>7 speckle tracking.mp. (3722)<br>8 STE.mp. (1905)<br>9 strain.mp. (385200)<br>10 deformation.mp. (36853)<br>11 mechanic*.mp. (390628)<br>12 torsion.mp. (22257)<br>13 twist.mp. (10298)<br>14 rotation.mp. (95760)<br>15 GLS.mp. (1508)<br>16 6 or 7 or 8 or 9 or 10 or 11 or 12 or 13 or 14 or 15 (963614)<br>17 Cardiovascular Diseases/ (128863)<br>18 cardiovascular disease*.mp. (220948)<br>19 Heart Failure/ (103286)<br>20 Heart failure.mp. (176882)<br>21 HF.mp. (35998)<br>22 Mortality/ (39168)<br>23 mortality.mp. (670200)<br>24 Death/ (16230)<br>25 death.mp. (691814)<br>26 Morbidity/ (27417)<br>27 morbidity.mp. (330859)<br>28 (cardi* adj3 (event* or outcome*)).mp. (66991)<br>29 17 or 18 or 19 or 20 or 21 or 22 or 23 or 24 or 25 or 26 or 27 or 28 (1690357)<br>30 predict*.mp. (1382430)<br>31 prognos*.mp. (734497)<br>32 30 or 31 (1934125)<br>33 exp Cohort Studies/ (1714168)<br>34 (cohort adj (study or studies)).mp. (310492)<br>35 (Follow up adj (study or studies)).mp. (604740)<br>36 Longitudinal.mp. (250750)<br>37 (observational adj (study or studies)).mp. (109821)<br>38 Epidemiologic studies/ (7611)

For peer review only

	39 (epidemiologic* adj (study or studies)).mp. (79971)<br>40 population based study.mp. (24755)<br>41 general population.mp. (85498)<br>42 communit*.mp. (535197)<br>43 33 or 34 or 35 or 36 or 37 or 38 or 39 or 40 or 41 or 42 (2539317)<br>44 5 and 16 and 29 and 32 and 43 (2940)
**EMBASE**	Database: Embase Classic+Embase <1947 to 2018 February 27><br>Search Strategy:<br>--<br>1 heart left ventricle function/ (39176)<br>2 left ventric*.mp. (332499)<br>3 LV.mp. (83461)<br>4 1 or 2 or 3 (353062)<br>5 echocardiography/ (178295)<br>6 exp speckle tracking echocardiography/ (3150)<br>7 speckle tracking.mp. (10536)<br>8 STE.mp. (4416)<br>9 strain.mp. (762166)<br>10 deformation.mp. (42935)<br>11 mechanic*.mp. (498347)<br>12 torsion.mp. (27446)<br>13 twist.mp. (12502)<br>14 rotation.mp. (109923)<br>15 GLS.mp. (3938)<br>16 5 or 6 or 7 or 8 or 9 or 10 or 11 or 12 or 13 or 14 or 15 (1553350)<br>17 cardiovascular disease/ (241943)<br>18 cardiovascular disease*.mp. (336057)<br>19 heart failure/ (212076)<br>20 Heart Failure.mp. (347509)<br>21 HF.mp. (60866)<br>22 mortality/ (722163)<br>23 cardiovascular mortality/ (29486)<br>24 all cause mortality/ (4807)<br>25 mortality.mp. (1271143)<br>26 death/ (284488)<br>27 death.mp. (1097060)<br>28 morbidity/ (309365)<br>29 morbidity.mp. (569914)<br>30 (cardi* adj3 (event* or outcome*)).mp. (110928)<br>31 17 or 18 or 19 or 20 or 21 or 22 or 23 or 24 or 25 or 26 or 27 or 28 or 29 or 30 (2824491)<br>32 predict*.mp. (1798936)<br>33 prognos*.mp. (982782)

For peer review only

	34	32 or 33 (2544622)
	35	longitudinal study/ (109328)
	36	longitudinal.mp. (297876)
	37	prospective study/ (426930)
	38	cohort analysis/ (346132)
	39	(Cohort adj (study or studies)).mp. (217636)
	40	(follow up adj (study or studies)).mp. (63815)
	41	(observational adj (study or studies)).mp. (172984)
	42	(epidemiologic* adj (study or studies)).mp. (100634)
	43	population based study.mp. (33254)
	44	general population.mp. (124629)
	45	communit*.mp. (641283)
	46	35 or 36 or 37 or 38 or 39 or 40 or 41 or 42 or 43 or 44 or 45 (1963334)
	47	4 and 16 and 31 and 34 and 46 (4098)

'adj3' is a proximity operator and indicates within three words;

'OR and AND' are Boolean operators;

'*' indicates truncation;

'mp.' means a keyword search of title, abstract, original title, name of substance word, subject heading word and keyword heading word.

For peer review only

Eligibility Form: Prognostic Implications of Left Ventricular Strain by Speckle-Tracking Echocardiography in Population-based Studies

**1. Reference details**				
Ref ID				
Reference citation				
First author				
Year of publication				
Title of the paper				
Type of publication	Paper	☐	Abstract	☐
Assessor's identifier	1	☐	2	☐
Date				
Contact details of authors				

**2. Study eligibility**			
Inclusion of the study	Yes ☐ No ☐	Unclear ☐	
**Eligibility criteria**	**Reason for exclusion (if excluded)**	Yes	No
Longitudinal (cohort) study	• Ineligible study design e.g. Cross-sectional, case-control, or CRT	☐	☐
General population, community/population-based samples/ unselected community-dwelling individuals	• Ineligible population e.g. patients with established CVD, not population-based, unrepresentative samples of the general population	☐	☐
LV strain measured by STE	• Ineligible exposure e.g. measured by MRI	☐	☐
	• Ineligible outcome (Different from outcomes considered in this review e.g. LV remodelling)	☐	☐
	• Duplicate	☐	☐
	• Reviews, abstracts, letters, or conference proceeding	☐	☐
	• Other (please specify): e.g. not in English or in a peer reviewed journal	☐	☐
Notes:			

**Additional information**
Notes:

DO NOT PROCEED IF PAPER EXCLUDED FROM REVIEW

For peer review only

Data Extraction Form: Prognostic Implications of Left Ventricular Strain by Speckle-Tracking Echocardiography in Population-based Studies

**3. Study details**		
Ref ID		
Study (cohort) name		
Study design	Longitudinal (cohort) study ☐	
Region/country		
Sample size		
Mean (range) age	Mean ± SD :	Range (IQR):
Sex	Male, n (%):	Female, n (%):
Ethnicity		
Follow-up duration		
Clinical variables (n, %)		
• Hypertension		
• Diabetes mellitus		
• Dyslipidaemia		
• Smoking status		
• Known CVD		
Dose the study include/mention a statement regarding the ethical approval, or adherence to an appropriate standard (such as the declaration of Helsinki)? Yes ☐ No ☐		

**4. Exposure details**			
Hardware		Software	
Procedure:	2D-STE ☐	3D-STE ☐	
Measured LV strain (direction)		Images obtained from	number of LV segments
Longitudinal:	Yes ☐ No ☐		
Circumferential:	Yes ☐ No ☐		
Radial:	Yes ☐ No ☐		
Others (specify):	Yes ☐ No ☐		
Number of sonographers used to perform the analysis:		Provide data on intra- or inter-observer variability or both?	Yes ☐ No ☐<br>Note:
Notes:			

**5. Outcomes' details**	
Type of outcomes used in this review	Outcomes reported in this study (n)
**Primary outcome(s)**<br>All-cause mortality	

For peer review only

**Secondary outcome (s)**<br>A. Composite **cardiovascular** (CV) end point includes: CV mortality, CHD events (MI, unstable angina, angina/ischemia requiring emergent hospitalization or revascularization), HF hospitalization, new onset AF, life threatening arrhythmia , recorded automatic implantable cardioverter defibrillator (AICD) shocks, stroke, transit ischemic attack, peripheral arterial disease with arterial revascularization procedure.	
B. Composite **cardiac** end point includes: CV mortality, CHD events, HF hospitalization, new onset AF, life threatening arrhythmia, recorded AICD shocks.	
Report an individual end point included in the composite cardiac or CV end point	Yes ☐ No ☐<br>List if yes:
Assessment of outcome	☐ independent blind assessment ☐ record linkage<br>☐ self-report ☐ others
Notes:	

**6. Available number of participants**		
		Number
Baseline sample size	Yes ☐ No ☐	
Excluded from the baseline	Yes ☐ No ☐<br>Reason if yes:	
Lost to follow-up	Yes ☐ No ☐	
Other losses	Yes ☐ No ☐	
Total number included in the analysis	Yes ☐ No ☐	
All subjects accounted for	Yes ☐ No ☐	
Notes:		

**7. Statistical analysis**	
Statistical method used	Cox proportional hazards regression Yes ☐ No ☐<br>Others if 'No':
Analysis includes males and	Yes ☐ No ☐

females	
Study reports results by sex	Yes ☐ No ☐
List all models used including unadjusted	

For peer review only

7. **Results of the association of interest**								
Exposure	Outcome	Group (M/F/All)	Total (n) included in the analysis	Events/non-events	Effect estimate	95% CI, SE, p	Unit	Adjustments (none if unadjusted)

For peer review only

NEWCASTLE - OTTAWA QUALITY ASSESSMENT SCALE - COHORT STUDIES (Amended)

Note: A study can be awarded a maximum of one star for each numbered item within the Selection and Outcome categories.

Selection

1) Representativeness of the cohort
   a) Truly representative of the average person in the community ❋
   b) Somewhat representative of the average person in the community (sampling described and designed to achieve adequately representative sample of the community)❋
   c) Selected group e.g. nurses, volunteers
   d) No description of the derivation of the cohort
2) Assessment of exposure reliability
   a) Include assessment of inter- and intra-observer variabilities ❋
   b) Include assessment of intra-observer variability ❋
   c) No description
3) Completeness of collection of potential confounders[1](i.e. CVD risk factors)
   a) Complete ❋
   b) Partially complete (≥ 5) ❋
   c) Partially complete (< 5)
   d) No description
4) Demonstration that the outcome of interest not present at start of study (and/or sensitively analysis)
   a) Yes ❋
   b) No

Outcome

1) Assessment of outcome
   a) Independent blind assessment ❋
   b) Record linkage ❋
   c) Self-report
   d) No description
2) Was follow-up long enough for outcomes to occur
   a) yes (an adequate follow up period for outcome of interest is a year) ❋
   b) no
3) Adequacy of follow up of cohorts
   a) Complete follow up - all subjects accounted for ❋
   b) Subjects lost to follow up unlikely to introduce bias - small number lost - > 80% follow up, or description provided of those lost ❋
   c) Follow up rate < 80% and no description of those lost
   d) No statement

The total quality score will be calculated by averaging the score of the two researchers with the range from 0 (lowest quality) to 7 (highest quality) and will be provided as a descriptive variable of the included studies.

1 Based on Framingham risk factors modified to include ethnicity: age, sex, blood pressure, antihypertensive medication (yes/no), total cholesterol, HDL cholesterol (or BMI), smoking, diabetes mellitus, and ethnicity.

For peer review only

Reporting checklist for protocol of a systematic review.

Based on the PRISMA-P guidelines.

Instructions to authors

Complete this checklist by entering the page numbers from your manuscript where readers will find each of the items listed below.

Your article may not currently address all the items on the checklist. Please modify your text to include the missing information. If you are certain that an item does not apply, please write "n/a" and provide a short explanation.

Upload your completed checklist as an extra file when you submit to a journal.

In your methods section, say that you used the PRISMA-P reporting guidelines, and cite them as:

Moher D, Shamseer L, Clarke M, Ghersi D, Liberati A, Petticrew M, Shekelle P, Stewart LA. Preferred Reporting Items for Systematic Review and Meta-Analysis Protocols (PRISMA-P) 2015 statement. Syst Rev. 2015;4(1):1.

		Reporting Item	Page Number
Identification	#1a	Identify the report as a protocol of a systematic review	1
Update	#1b	If the protocol is for an update of a previous systematic review, identify as such	n/a
	#2	If registered, provide the name of the registry (such as PROSPERO) and registration number	7
Contact	#3a	Provide name, institutional affiliation, e-mail address of all protocol authors; provide physical mailing address of corresponding author	1
Contribution	#3b	Describe contributions of protocol authors and identify the guarantor of the review	13
	#4	If the protocol represents an amendment of a previously completed or published protocol, identify as such and list changes; otherwise, state plan for documenting important protocol amendments	n/a

For peer review only

Sources	#5a	Indicate sources of financial or other support for the review	13
Sponsor	#5b	Provide name for the review funder and / or sponsor	n/a
Role of sponsor or funder	#5c	Describe roles of funder(s), sponsor(s), and / or institution(s), if any, in developing the protocol	n/a
Rationale	#6	Describe the rationale for the review in the context of what is already known	4, 5, 6
Objectives	#7	Provide an explicit statement of the question(s) the review will address with reference to participants, interventions, comparators, and outcomes (PICO)	5, 6
Eligibility criteria	#8	Specify the study characteristics (such as PICO, study design, setting, time frame) and report characteristics (such as years considered, language, publication status) to be used as criteria for eligibility for the review	7, 8, 9
Information sources	#9	Describe all intended information sources (such as electronic databases, contact with study authors, trial registers or other grey literature sources) with planned dates of coverage	9, 10
Search strategy	#10	Present draft of search strategy to be used for at least one electronic database, including planned limits, such that it could be repeated	9 (S file 1)
Study records - data management	#11a	Describe the mechanism(s) that will be used to manage records and data throughout the review	9
Study records - selection process	#11b	State the process that will be used for selecting studies (such as two independent reviewers) through each phase of the review (that is, screening, eligibility and inclusion in meta-analysis)	9, 10
Study records - data collection process	#11c	Describe planned method of extracting data from reports (such as piloting forms, done independently, in duplicate), any processes for obtaining and confirming data from investigators	10, 11
Data items	#12	List and define all variables for which data will be sought	10, 11

For peer review only

		(such as PICO items, funding sources), any pre-planned data assumptions and simplifications	
Outcomes and prioritization	#13	List and define all outcomes for which data will be sought, including prioritization of main and additional outcomes, with rationale	8
Risk of bias in individual studies	#14	Describe anticipated methods for assessing risk of bias of individual studies, including whether this will be done at the outcome or study level, or both; state how this information will be used in data synthesis	11, 12
Data synthesis	#15a	Describe criteria under which study data will be quantitatively synthesised	12
	#15b	If data are appropriate for quantitative synthesis, describe planned summary measures, methods of handling data and methods of combining data from studies, including any planned exploration of consistency (such as I2, Kendall's τ)	11, 12
	#15c	Describe any proposed additional analyses (such as sensitivity or subgroup analyses, meta-regression)	11, 12
	#15d	If quantitative synthesis is not appropriate, describe the type of summary planned	12
Meta-bias(es)	#16	Specify any planned assessment of meta-bias(es) (such as publication bias across studies, selective reporting within studies)	12
Confidence in cumulative evidence	#17	Describe how the strength of the body of evidence will be assessed (such as GRADE)	n/a (justification provided in p11)

The PRISMA-P checklist is distributed under the terms of the Creative Commons Attribution License CC-BY 4.0. This checklist can be completed online using https://www.goodreports.org/, a tool made by the EQUATOR Network in collaboration with Penelope.ai

For peer review only
